# A comprehensive review for artificial intelligence on neuroimaging in rehabilitation of ischemic stroke

**DOI:** 10.3389/fneur.2024.1367854

**Published:** 2024-03-28

**Authors:** Zijian Zhao, Yuanyuan Zhang, Jiuhui Su, Lianbo Yang, Luhang Pang, Yingshan Gao, Hongbo Wang

**Affiliations:** ^1^Rehabilitation Center, ShengJing Hospital of China Medical University, Shenyang, Liaoning Province, China; ^2^Department of Orthopedics, Haicheng Bonesetting Hospital, Haicheng, Liaoning Province, China; ^3^Department of Reparative and Reconstructive Surgery, The Second Hospital of Dalian Medical University, Dalian Liaoning Province, China; ^4^Department of Radiology, Shengjing Hospital of China Medical University, Shenyang, Liaoning Province, China; ^5^Department of Radiology, Shengjing Hospital of China Medical University, Shenyang, Liaoning Province, China

**Keywords:** ischemic stroke, rehabilitation, artificial intelligence, MRI, CT

## Abstract

Stroke is the second leading cause of death worldwide, with ischemic stroke accounting for a significant proportion of morbidity and mortality among stroke patients. Ischemic stroke often causes disability and cognitive impairment in patients, which seriously affects the quality of life of patients. Therefore, how to predict the recovery of patients can provide support for clinical intervention in advance and improve the enthusiasm of patients for rehabilitation treatment. With the popularization of imaging technology, the diagnosis and treatment of ischemic stroke patients are often accompanied by a large number of imaging data. Through machine learning and Deep Learning, information from imaging data can be used more effectively. In this review, we discuss recent advances in neuroimaging, machine learning, and Deep Learning in the rehabilitation of ischemic stroke.

## 1 Introduction

### 1.1 Epidemiology of ischemic stroke

Stroke stands as the second leading cause of global mortality and a primary contributor to disability and cognitive impairment (1). Stroke is classified into ischemic stroke and hemorrhagic stroke. Among these, ischemic stroke prevails. Approximately 9.5 million cases of ischemic stroke were reported globally in 2016 (2). In addition, 2.7 million people succumbed to ischemic stroke each year worldwide (3). Thromboembolism remains the leading cause of most ischemic strokes, primarily attributed to large artery atherosclerosis and cardiac conditions, particularly atrial fibrillation (4).

### 1.2 The application of artificial intelligence in the field of stroke

Artificial Intelligence (AI) technology is a rapidly advancing field. In the realm of brain diseases, AI is widely employed for the detection, segmentation, classification, and identification of large vessel occlusion (LVO) in both hemorrhagic and ischemic strokes. Tang et al. (5) proposed a computer-aided detection scheme that detects early-stage ischemic strokes with small lesions through image feature analysis. The use of this method was found to improve stroke detection by healthcare professionals. The diagnosis of LVO is particularly crucial for selecting patients suitable for mechanical thrombectomy. An artificial neural network (ANN) algorithm developed by Chen et al. (6) demonstrated a high predictive accuracy of 0.820 for LVO, surpassing other prehospital prediction models. Additionally, research suggests that radiomics scores serve as independent prognostic indicators for the outcomes of acute ischemic stroke (7). Pfaff et al. (8) indicated that the e-ASPECTS software can be utilized to predict adverse outcomes after mechanical thrombectomy. Furthermore, survivors of strokes often experience upper limb motor deficits and achieve limited functional recovery within 6 months post-stroke. Many studies suggest the widespread application of robots in assisting patients with motor function rehabilitation (9). The utilization of artificial intelligence for the accurate analysis of medical images and clinical data, enabling rapid and precise identification of cerebrovascular disease types and etiologies. This facilitates the development of personalized treatment and rehabilitation plans, ultimately leading to improved patient survival rates and quality of life (10).

#### 1.2.1 Related research trends

**Figure 1:**
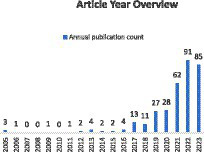
Number of published studies on ischemic stroke in the past 15 years.

As depicted in Figure 1, articles related to ischemic stroke have shown an upward trend in the past 15 years. With the rapid development and widespread application of Artificial Intelligence in the field of medicine, research in this area has experienced an explosive growth in the last 3 years. Furthermore, it is evident that there are only 337 articles specifically focused on evaluating the prognosis or rehabilitation of ischemic stroke, constituting a small fraction of the overall literature. In comparison to prognosis prediction, more studies are concentrated on the detection of ischemic stroke itself.

#### 1.2.2 Detection modalities

**Figure 2:**
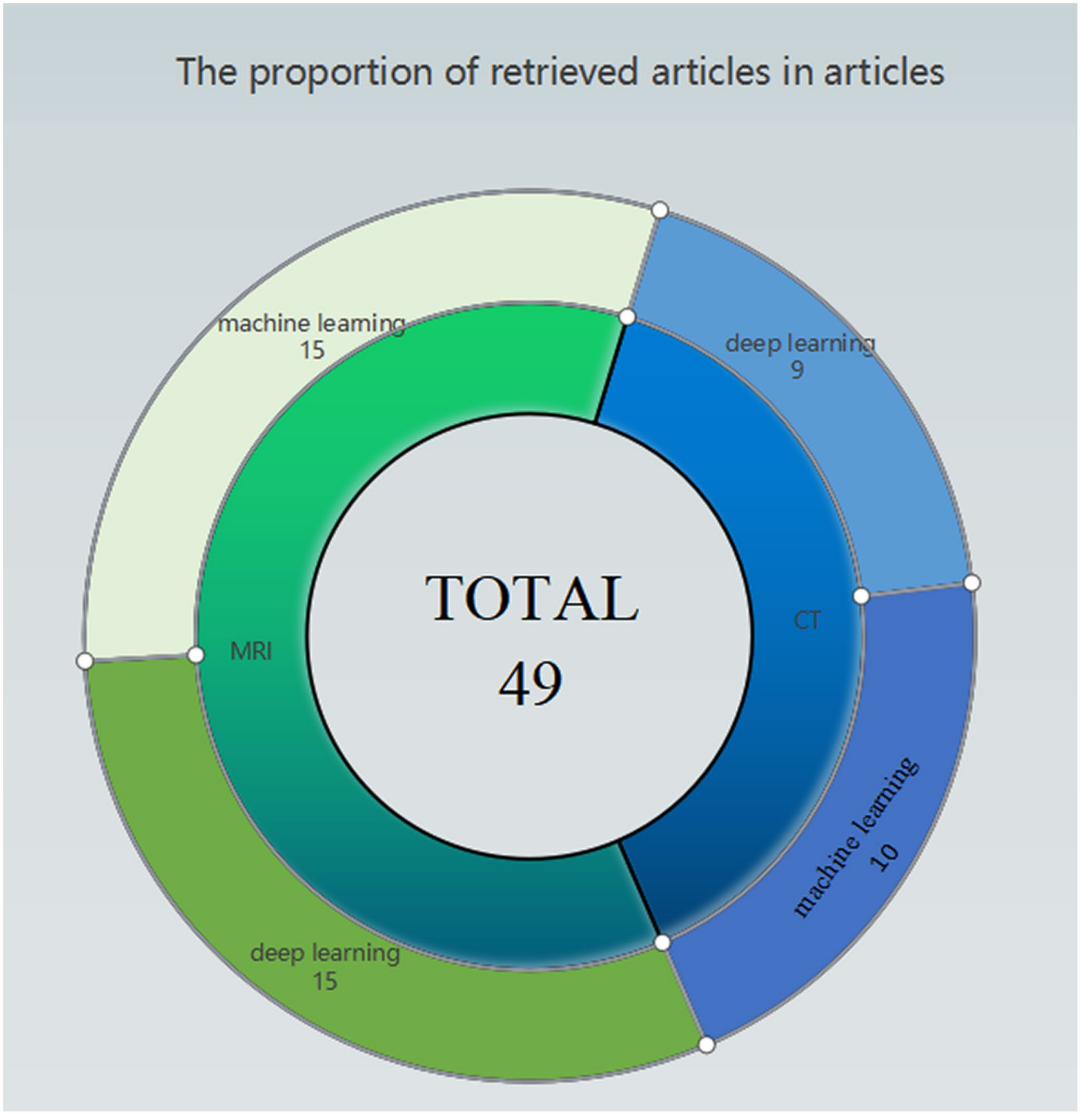
Pie chart of classes of studies included in this review.

As illustrated in Figure 2, articles pertaining to the application of Artificial Intelligence in ischemic stroke have been summarized and categorized based on different data types. It is noteworthy that the prediction of ischemic stroke using medical imaging data emerges as the most prominent area of focus. A significant proportion of articles also revolves around prognostic evaluations based on the mRS scale.

### 1.3 Article retrieval

**Figure 3:**
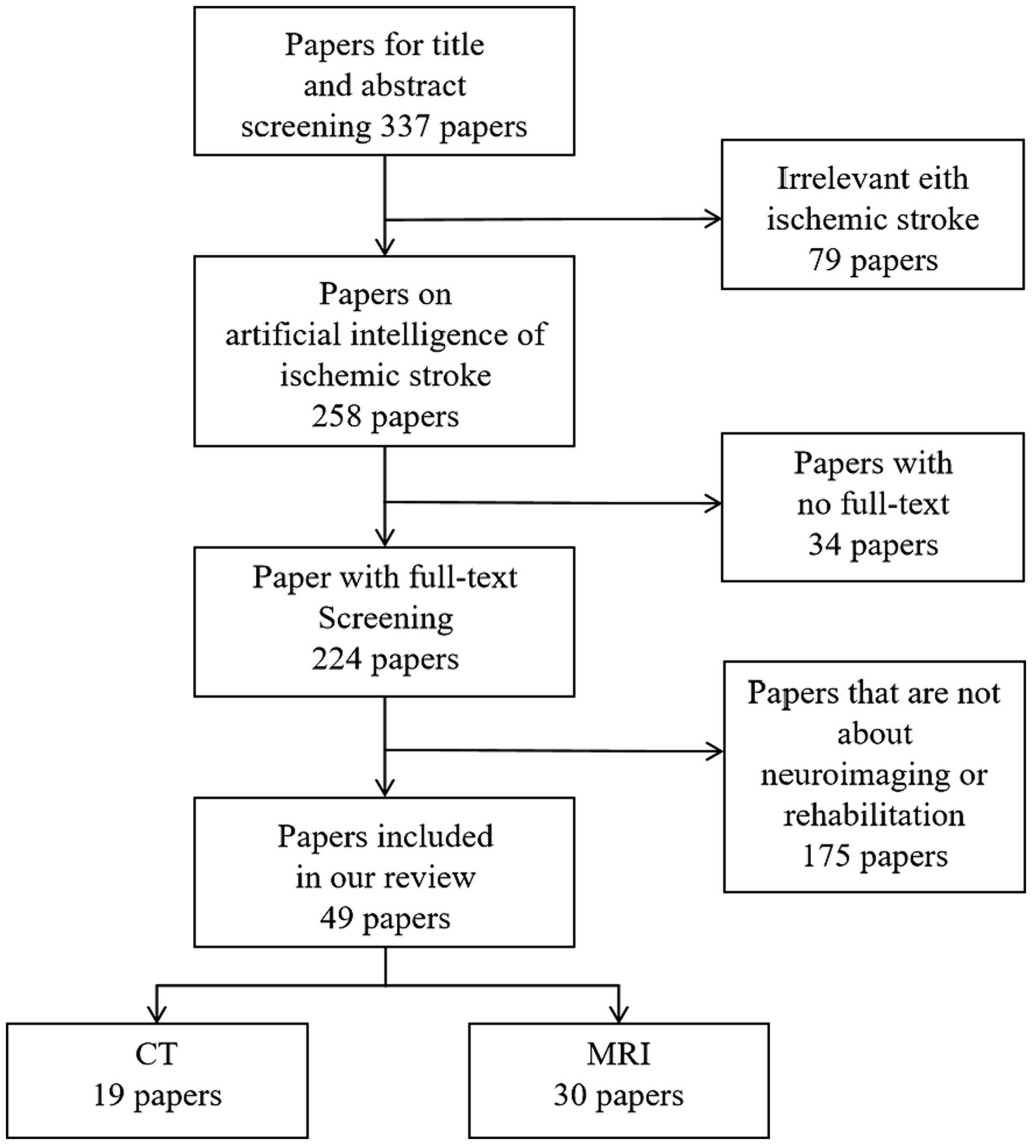
Flow chart of paper selection process.

To gather relevant papers for our study, a comprehensive search strategy was devised, employing various combinations of the following keywords: “stroke,” “ischemic stroke,” “prognosis,” “rehabilitation,” “Deep Learning,” “machine learning,” and “Artificial Intelligence.” Considering technological advancements and updates, we restricted the publication timeframe to the past 15 years. Using PubMed, Embase, Web of Science, and the Cochrane Library for the search, we initially included all articles reporting on ischemic stroke patients. This yielded 337 articles. After a meticulous review of abstracts and full texts, we first excluded articles not aligned with the research theme, then eliminated those without full texts, and finally removed articles not utilizing Artificial Intelligence for predicting ischemic stroke prognosis or rehabilitation. The selected literature focused on key technologies, resulting in a final set of 49 articles on Artificial Intelligence predictions of ischemic stroke prognosis or rehabilitation as our references. The paper selection process is illustrated in Figure 3.

### 1.4 The purpose of this article

While existing literature has summarized the research progress of AI in the field of ischemic stroke, most of it has focused primarily on pre-treatment prediction. For instance, Sheth et al. (11) provided an overview of common machine learning methods and their applications in detecting large vessel occlusion, intracranial hemorrhage, and infarct lesions. Soun et al. (12) systematically introduced AI methods in imaging and available public and commercial platforms, summarizing the applications of AI in acute stroke detection and prediction. Despite the detailed content, it did not address the prognosis of stroke patients, which is precisely the focal point of concern for most clinical professionals and patients. Ragoș et al. (13) summarized the evaluation of ischemic stroke outcomes using MRI radiomics and predictive models. However, relying solely on radiomic evaluation is too narrow, and the described imaging methods are not comprehensive enough. Shafaat et al. (14) evaluated the efficacy of machine learning in predicting the prognosis of ischemic stroke patients, overlooking research using methods such as Deep Learning and other AI techniques in this direction. Rüdiger von Kummer and colleagues summarized the progress of CT and MRI brain imaging technologies in acute ischemic stroke (15). This review focuses exclusively on CT/CTA and MRI/MRA imaging. With the advancement of Artificial Intelligence, many new technologies with greater potential applications have emerged. For instance, there is an urgent need to investigate the application value of CTP in ischemic stroke. Therefore, this article aims to systematically evaluate the potential applications of Artificial Intelligence in predicting the prognosis of ischemic stroke patients in the field of neuroimaging, primarily using CT and MR imaging.

## 2 Progress in predicting the rehabilitation of ischemic stroke based on artificial intelligence of neuroimaging

### 2.1 Radiological manifestations of ischemic stroke

The current diagnostic approach for stroke relies on CT and MRI imaging. MRI has higher sensitivity and specificity for diagnosing ischemic stroke, but due to factors such as longer imaging times and higher costs, CT-based imaging techniques remain the preferred method for diagnosing ischemic stroke. Vascular imaging through CT and MRI aids in identifying the extent of ischemia and the location of arterial occlusion. Furthermore, the results of radiological examinations play a crucial role in determining the treatment approach for ischemic stroke patients, providing support for interventions like thrombolysis and thrombectomy.

### 2.2 Ischemic stroke treatment

Treatment methods include intravenous thrombolysis, intra-arterial thrombolysis, mechanical thrombectomy, etc. (16). Currently, thrombolytic therapy is the most used treatment for ischemic stroke. The basic principle involves the activation of plasminogen into plasmin by the binding of the thrombus to fibrin, and plasmin breaks down fibrinogen and fibrin, dissolving the thrombus and allowing reperfusion of the ischemic brain (17). Intravenous thrombolysis is established for patients within 4.5 h of stroke onset. If symptoms appear within 6–8 h, mechanical vascular recanalization through stent retriever and/or thrombus aspiration is recommended.

### 2.3 Methods for evaluating prognosis in ischemic stroke

#### 2.3.1 Modified Rankin scale

The modified Rankin Scale (mRS) is the most widely used measure for assessing the outcomes of acute ischemic stroke in research, clinical trials, and national and local quality improvement registries. It reflects the quality of life as assessed by both patients and healthcare professionals. In certain situations, an mRS score of 5 (bedridden, requiring constant care, and severe disability) is considered more severe than an mRS score of 6 (death) (18). However, studies indicate that among patients who have undergone hemicraniectomy, over 50% experience moderate to severe disability postoperatively, yet remain satisfied with their life status (19). In clinical practice, achieving an mRS score between 0 and 2 (indicating functional independence) is generally considered a treatment success. Additionally, monitoring changes in the degree of disability is crucial. For instance, recovering to a mRS score of 3 is considered better than death or the need for nursing home care (mRS score of 5). Such transitions in disability levels can significantly reduce healthcare costs (4).

#### 2.3.2 Muscle strength assessment

The severe impairment of limb function caused by stroke significantly affects the quality of life of stroke patients. The recovery of post-stroke patients is correlated with the location and size of the infarction. Among them, the primary issue is the motor dysfunction caused by damage to the corticospinal tract and brain motor centers (20). Therefore, improving the muscle strength of patients is an important indicator for assessing the quality of life of stroke patients. Li et al. developed a rehabilitation program for the self-care ability of Acute Ischemic Stroke (AIS) patients based on six levels of commonly used muscle pain assessment methods in clinical practice. This program showed improvement in patients’ muscle strength, quality of life, and self-care ability by the third month (21). Fugl-Meyer and others devised a measurement method for functional recovery after cerebrovascular accidents, utilizing an accumulated numerical scoring system. They conducted a 1-year follow-up study on hemiplegic patients, ultimately achieving quantitative assessment of patients’ physical functions. This made the scale suitable for statistical analysis in both research and clinical settings (22).

#### 2.3.3 Imaging-based rehabilitation assessment

The degree of early ischemic changes on CT is correlated with stroke severity scores, such as NIHSS and serves as a predictive indicator of clinical outcomes. CTA and CT Perfusion (CTP) imaging are methods used to determine the collateral circulation blood flow status in patients, aiding in the selection of suitable candidates for intra-arterial treatment (23, 24). Patients with poor CTA collateral status tend to have a poorer prognosis even after reperfusion therapy (25). Early improvement in neurological function can often lead to a favorable prognosis, even without additional reperfusion therapy following intravenous tPA administration (26). The extent of collateral circulation may also help in selecting patients who benefit from reperfusion therapy beyond the current time windows for both intravenous and intra-arterial treatments (27). Several studies indicate that positive results in DWI are associated with specific clinical features, including longer duration of symptoms, motor deficits, aphasia, and large vessel occlusion on Magnetic Resonance Angiography (MRA) (28–30). Importantly, research suggests that positive DWI results play a crucial role in prognosis. Specifically, these studies show a higher risk of recurrent ischemic events in patients with abnormal findings on DWI scans during transient ischemic attacks (TIA) compared to those without abnormalities (30).

### 2.4 Artificial intelligence-based prediction of stroke prognosis using CT

Distinguishing ischemic stroke from hemorrhagic stroke remains challenging solely through clinical means. Brain CT imaging becomes pivotal in aiding differential diagnosis. Non-enhanced CT scans swiftly and intuitively assist clinicians by showcasing distinct radiological features between ischemic and hemorrhagic strokes. Apart from aiding in the differential diagnosis of hemorrhagic stroke, non-enhanced CT scans are also useful in evaluating the extent of early ischemic damage. Early non-enhanced CT signs of ischemia encompass sulcal effacement and decreased attenuation (31), leading to a loss of gray-white matter differentiation. In some patients, early signs of cerebral ischemic changes, such as loss of gray-white matter differentiation, is suffice for diagnosing ischemic stroke. However, these changes are subtle, particularly within the initial few hours of stroke onset, making the loss of gray-white matter differentiation challenging to discern (4). In general, non-enhanced CT scans exhibit a sensitivity of approximately 52% in detecting substantial ischemic parenchymal changes (32).

CT angiography (CTA) is a perfusion contrast tracking technique capable of displaying major vessels from the aorta to the cranial apex within 15 s (33). CTA boasts superior spatial resolution, surpassing most MRI vascular imaging sequences. It reveals the location and size of occlusive thrombi and provides information regarding collateral blood supply to the ischemic area. CTA exhibits a sensitivity of 95–99% in detecting significantly narrowed or occluded vessels (34). It can determine occlusion locations within 24 h of symptom onset and aid in deciding the suitability for mechanical thrombectomy (35). Besides routine CTA, multiphase CTA, involving imaging before and after contrast passing through different arteries and veins in the brain, can assess collateral circulation status, aiding in evaluating patients suitable for mechanical thrombectomy (36). CTA facilitates assessment of cerebral blood flow and identification of tissue areas at risk of infarction and potential recovery zones (37).

CT perfusion imaging (CTP) is a medical imaging technique that utilizes continuous CT scans of the region of interest to observe changes in contrast agent concentration, thus obtaining time-density curves of the region of interest, indirectly reflecting changes in organ perfusion (38). CTP offers advantages of rapid imaging, effectively and quantitatively reflecting changes in local tissue blood perfusion, and is widely used clinically for the examination of cerebral ischemia (39). Therefore, CTP enables the evaluation of ischemic tissue, aiding in the timely restoration of normal blood supply.

**Figure 4:**
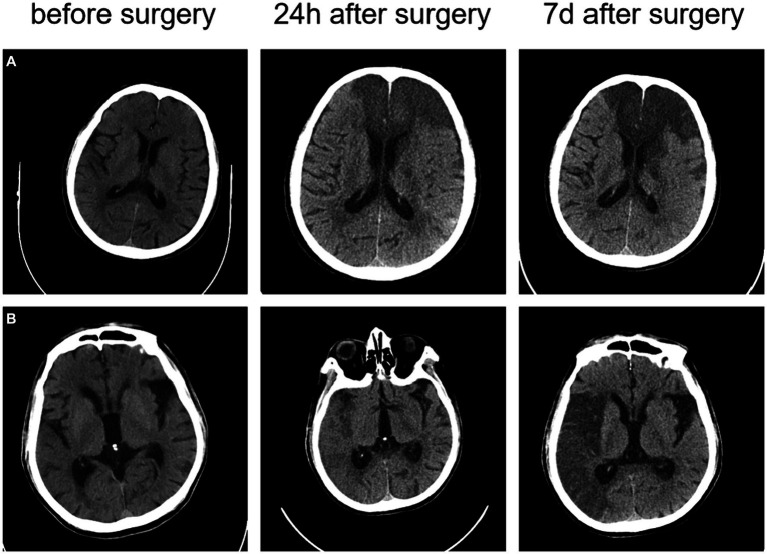
The CT images of two ischemic stroke patients undergoing thrombectomy **(A)** a 73-year-old male patient with an NIHSS score of 24 at the onset; and **(B)** a 78-year-old male patient with an NIHSS score of 14 at the onset.

The head CT scan is the preferred diagnostic method for the initial assessment of suspected stroke patients, as shown in Figure 4. Over time, CT can capture the difference in the image of the patient’s brain. CT scans are widely available, cost-effective, and provide rapid results. Modern CT scanners can examine the entire brain in less than 1 s. However, it is difficult to differentiate between acute ischemic stroke (AIS) and intracerebral hemorrhage (ICH) based solely on clinical presentation. In CT images, acute ICH is characterized by a higher density shadow that appears brighter than normal brain tissue.

#### 2.4.1 The application of machine learning in stroke

In 2019, Xie et al. incorporated CT, CTA, and perfusion CT data from 512 patients with acute ischemic stroke. Seven binomial GBM and XGB prediction models were developed using 23 features at admission, to predict patients’ mRS scores at 90 days. After adding the 24-h NIHSS score, the results of the study showed that the predictive performance of the models was significantly improved with the addition of the 24-h NIHSS score, with AUCs ranging from 0.794 to 0.873 for the XGB model and 0.811 to 0.866 for the GBM model. The conclusions of the study suggest that machine learning can be used to predict the outcome of rehabilitation in stroke patients, with initial imaging information is sufficient, the inclusion of 24-h information improves accuracy, and consideration of recanalization status helps assess treatment risk and benefit (40).

In 2020, Wen et al. incorporated clinical information and NCCT and CTA data from January 30, 2017, to January 2, 2019. These data were obtained within 24 h after symptom onset in patients with MCA territory infarction. Their aim was to develop a model based on radiomic features to predict the development of malignant MCA infarction (mMCAi) in stroke patients. Patients were randomly divided into a training group (*n* = 87) and a validation group (*n* = 39). A total of 396 texture features were extracted from each NCCT image of 126 patients. Using least absolute shrinkage and selection operator regression analysis to reduce the feature dimensions, precise radiomic features were constructed based on the remaining texture features. Subsequently, a radiomic feature model was built using multivariate logistic regression, and its performance was evaluated using AUC. Decision curve analysis (DCA) was employed to assess the clinical efficiency of radiomic features in predicting mMCAi by calculating the net benefit within a threshold probability range. They then developed a model combining radiomic features and the Alberta Stroke Program Early CT Score (ASPECTS) based on NCCT to predict mMCAi. The predictive model demonstrated excellent performance, with AUCs of 0.917 and 0.913 for the training and validation sets, respectively. Furthermore, DCA validated the clinical effectiveness of the predictive model in distinguishing mMCAi and non-mMCAi patients within a threshold probability range of 0.067–1 in the training set and 0.046–1 in the validation set (41).

In 2021, Cheng et al. included CT and CTA data from 135 patients with large vessel occlusive stroke who underwent reperfusion therapy between 2015 and 2019. The aim was to explore the correlation between different CT-ASPECTS (Alberta Stroke Program Early CT Score) methods, follow-up CT-ASPECTS, and prognosis. Researchers calculated the relative differences in Hounsfield Units (HU) between different regions of the ischemic hemisphere and the average HU of the contralateral hemisphere, expressed as a percentage difference. The NCCT, CTA-arterial, and CTA-venous datasets were evaluated in a random order and validated by two expert readers after correctional segmentation. ROC curve analysis was used to assess the ability of different CT-ASPECTS patterns to identify patients with favorable outcomes. Researchers found that CTA-venous-ASPECTS was almost perfectly correlated with follow-up CT-ASPECTS, outperforming other CT examinations. The 90-day mRS scores were significantly associated with CTA-venous-ASPECTS. ROC analysis defined the optimal accuracy and cutoff points for parameters related to the 90-day mRS score. The results indicated that CTA-venous-ASPECTS had the highest area under the curve (AUC: 0.82; 95%CI: 0.75–0.89; *p* < 0.001). This study suggests that CTA-venous-ASPECTS is almost perfectly correlated with the final infarct size and significantly associated with the 90-day mRS score (42).

In 2022, Potreck et al. included 136 stroke patients with major segment occlusion of MCA that occurred between March 2015 and December 2019. Two raters assessed ASPECTS on acute and follow-up NCCT, and a machine-learning algorithm evaluated the ASPECTS scale on NCCT (e-ASPECTS). A third radiologist used the MCA territory collateral score (also known as the Tan scoring system) to assess collateral status on the CT angiogram. The results indicate that inter-rater reliability depends on the duration of stroke symptoms in patients (OTI), with lower reliability observed in the hyperacute group, yielding ICC = 0.54, while higher reliability is seen in groups with longer time windows, yielding ICC = 0.74. The consistency between acute and follow-up ASPECTS improves with prolonged time, and there is a negative correlation between OTI time and ASPECTS. The collateral status serves as a predictor for favorable clinical outcomes, especially in hyperacute stroke. In conclusion, the accuracy and reliability of NCCT-ASPECTS are influenced by time, and collateral status on CT angiography may enhance the prediction of clinical outcomes (43).

In 2022, a study included data from 39 patients with AIS caused by LVO and poor reperfusion after mechanical thrombectomy (MT) from a stroke database between January 2015 and December 2019. The multimodal stroke protocol included non-contrast-enhanced computed tomography (NECT), CTP, and CTA in sequence. The ASPECTS score was used to assess whether early ischemic changes were present on baseline NECT. Three different automated perfusion software solutions (A: RAPID, B: Brainomix e-CTP, C: Syngo.via) were used to assess poor reperfusion. Low-perfusion volumes (HV) with Tmax >6 s were compared with the final infarct volume (FIV) on follow-up CT after futile reperfusion at 36–48 h. The study divided patients into high and low Hyperintense Rim (HIR) groups based on the median ratio of low-perfusion intensity (HIR, tissue volume ratio for Tmax >10 s and Tmax >6 s). Subgroup analyses of FIV (feature importance value) were conducted for favorable and unfavorable HIR. HIR was correlated with baseline clinical and outcome parameters using Pearson correlation. The study found a good correlation between HV and FIV with no significant difference. However, in cases with infarct volumes exceeding 150 mL, the performance of automated software solutions often declined. Subgroup analysis showed that patients with HIR ≥ 0.6 typically had underestimated FIV. However, in the subgroup with favorable HIR, there was a trend of overestimating FIV. Software packages A and B showed good correlation between HV and FIV with no significant difference, while only software package C significantly overestimated FIV. The mRS score of 0–3 at 3 months was significantly higher in the favorable HIR group than in the unfavorable group. Lower HIR was associated with a higher Alberta Stroke Program Early CT Score (ASPECTS). In conclusion, the performance of automated perfusion software solutions in predicting FIV after futile reperfusion was good, with a decrease in accuracy for large infarcts exceeding 150 mL. However, FIV may be significantly overestimated or underestimated depending on HIR, and the Syngo software package showed the widest range of performance (44).

In 2023, Xiang et al. collected one-stop CTP imaging data from 54 patients with AIS at Handan Central Hospital. The data included non-contrast CT scans, CTA, T_max_ maps, and CBF maps before and after conservative treatment and mechanical thrombectomy. Among the 54 patients, 15 underwent both CTP and MRI examinations. The post-processing method involved transferring CTP data to Artificial Intelligence software to obtain pseudo-colored images, ischemic core volume, and areas of abnormal perfusion. Additionally, Artificial Intelligence software was utilized to acquire intracranial arterial CTA images. The results revealed that patients treated with mechanical thrombectomy guided by CTP imaging had significantly improved NIHSS scores compared to the conventional treatment group, and this difference was statistically significant (*p* < 0.005). In one case assessed with Artificial Intelligence-assisted CTP imaging, the ischemic core volume was greater than that displayed by DWI, while in the remaining 13 patients; the ischemic core volume was smaller than the DWI-displayed ischemic core volume. The team concluded that mechanical thrombectomy guided by CTP imaging can extend the treatment window for AIS-LVO patients. AI-assisted CTP diagnosis can facilitate rapid assessments independent of radiologists, but it may pose challenges in determining the ischemic core volume (45).

In 2023, Weng et al. included 97 stroke patients. The team extracted vascular structural features from CTA images and stroke location features from DWI images to comprehensively characterize the lesions. The 97 cases were randomly divided into a cross-validation set, independent test set 1, and independent test set 2 for model validation. The results showed that the proposed model achieved good predictive performance on two independent test sets, with classification accuracies of 85.19 and 81.25%, respectively (46).

In 2023, Zhang et al. collected clinical data and NCCT images from 240 patients with AIS. Using 3D Slicer, they manually segmented the infarct lesions and performed feature extraction on CT images and regions of interest (ROI). After normalizing clinical and radiological features, surplus features were eliminated using the Kruskal-Wallis test. Through triple cross-validation and grid search, the research team selected the optimal hyperparameters for the Support Vector Machine (SVM) model. The dataset was divided into 3-fold in each of the three cross-validation runs, forming three prediction models. The average performance metrics for these three models included accuracy, sensitivity, specificity, F1 score, and AUC. After an in-depth analysis of 1,454 texture features extracted from NCCT images of 240 AIS patients, it was found that the classification model integrating clinical and radiomic data performed the best, with an AUC of 0.857, accuracy of 84.8%, and sensitivity of 93.8%. In comparison, models using only clinical or radiomic features showed lower performance with AUCs of 0.705 and 0.643, respectively. These study results suggest that integrated models combining multiple types of data are more reliable in predicting clinical outcomes for AIS patients (47).

In 2023, Brugnara et al. conducted a study on acute ischemic stroke patients undergoing imaging examinations and EVT. They utilized e-ASPECTS (Brainomix) for automatic assessment of ASPECTS on 1 mm slices, and visual inspection was conducted by experienced radiologists (AE, with 2 years of experience) and committee-certified neuroradiologists (UN, with 8 years of clinical experience). Statistical analyses were performed using Logistic regression and ordinal Logistic regression. Model performance was evaluated through ROC curves, and the significance of differences between models was assessed using the DeLong test. Machine learning model performance was assessed through random forest variable importance. In the entire study cohort, 38% of patients exhibited favorable clinical outcomes, while 26% experienced adverse outcomes at 90 days. Multivariate regression model results indicated that cortical atrophy was independently predictive of favorable clinical outcomes. The predictive performance of the machine learning model significantly outperformed other models, achieving an AUC of 0.775. Further analysis validated the importance of cortical atrophy across different models. The study results suggest that cortical atrophy is an independent predictor of clinical prognosis in acute ischemic stroke patients (48).

In 2023, Shen et al. included 44 consecutive patients with AIS who underwent endovascular treatment. Clinical data, including baseline mCTA, mRS, and follow-up MRI after treatment, were collected. They utilized a multi-scale three-dimensional CNN, inputting NCCT, arterial phase peak CTA, and CTA+ images. The F-STROKE software was used to calculate subsequent infarct core (IC) volume based on DWI. Data analysis was conducted using SPSS and MedCalc software. Among the 44 AIS patients receiving endovascular treatment, 61.4% achieved a favorable outcome. The NIHSSpre at admission and mCTA-estimated IC volume were independently correlated with the functional outcome of AIS patients after mechanical thrombectomy. Patients with a favorable prognosis had lower NIHSSpre and smaller mCTA-estimated IC volume (20.3 ± 12.2 vs. 43.9 ± 23.5, *p* = 0.001), and a higher proportion of good collateral status (66.7 vs. 22.4%, *p* = 0.016). The integrated model showed the best performance, with an area under the ROC curve of 0.874. The mean onset-to-door time (ODT) and door-to-puncture time (DPT) were 75.6 and 16.3 min, respectively, with a successful reperfusion rate of 17.7%. Bland–Altman plots and intraclass correlation coefficient (ICC) assessment indicated an acceptable level of consistency between mCTA-estimated IC volume and follow-up IC volume. The optimal threshold for predicting performance was mCTA-estimated IC volume ≤ 40.3 mL. The study also focused on the handling of hemorrhagic transformation (HT) regions. Deep Learning techniques were employed to extract volume data from mCTA. The mCTA-estimated IC volume may have potential value in predicting follow-up infarct and clinical outcomes in AIS patients treated with endovascular therapy (49).

The main information of the above included literatures is shown in Table 1.

**Table 1:** Summary of papers on machine learning for CT on rehabilitation of Ischemic stroke.

**PMID**	30354266	32733197	34392005	34709408	35645395
**YEAR**	2019	2020	2021	2022	2022
**LEARNING APPROACH**	machine learning	machine learning	machine learning	machine learning	machine learning
**PRIMARY AUTHOR**	Xie Y	Wen X	Cheng X	A Potreck	Iris Muehlen
**DISEASE**	Acute Ischemic Stroke	Malignant middle cerebral artery infarction (mMCAi)	large-vessel occlusion stroke	Acute Stroke Symptom-onset	Large Vessel Occlusive Stroke and Poor Revascularization
**DATA VOLUME**	CT, CT angiography (CTA), and perfusion CT data from 512 patients.	A total of 396 texture features were extracted from each NCCT image from the 126 patients	135 patients undergoing reperfusion therapy.	136 patients with stroke involving occlusion of the main segment of the Middle Cerebral Artery (MCA)	39 patients underwent mechanical thrombectomy (MT) due to acute ischemic stroke (AIS) caused by anterior circulation large vessel occlusion (LVO) and impaired blood flow reconstruction.
**DATA TYPE**	Basis of Imaging, Demographic, and Clinical Information	NCCT、CTA	different CT modalities	NCCT	NECT、CTP、CTA
**METHODS**	Gradient Boosting Machine	model based on the radiomics signature and Alberta Stroke Program Early CT Score (ASPECTS) based on NCCT	Automated ASPECTS for multi-modality CT	NCCT-ASPECTS	Three Automated Perfusion Software Applications
**RESULTS**	In predicting mRS greater than 2, XGB and GBM have AUCs of 0.746 and 0.748, respectively. After incorporating the 24-hour NIHSS score, XGB's AUC increases to 0.884, and GBM's AUC increases to 0.877. Reperfusion status has a certain impact on predictions; XGB's AUC increases to 0.807 in non-reperfused patients but decreases to 0.670 in reperfused patients, while GBM's AUC increases to 0.781 in non-reperfused patients but decreases to 0.655 in reperfused patients. For predicting mRS greater than 0, XGB's AUC ranges from 0.794 to 0.873, and GBM's AUC ranges from 681.1 to 762.3. Considering the 24-hour NIHSS score, XGB's AUC ranges from 0.794 to 0.873, and GBM's AUC ranges from 0.811 to 0.866.	Their predictive model exhibits outstanding performance, with AUCs of 0.917 and 0.913 for the training and validation sets, respectively. Additionally, Decision Curve Analysis (DCA) validated the clinical effectiveness of the predictive model in distinguishing between mMCAi and non-mMCAi patients, with probability threshold ranges of 0.067–1 in the training set and 0.046–1 in the validation set.	Researchers found a nearly perfect correlation between CTA-venous-ASPECTS and follow-up CT-ASPECTS, which outperformed other CT scans. The 90-day Modified Rankin Scale (mRS) scores were significantly associated with CTA-venous-ASPECTS. ROC analysis defined the optimal accuracy and cutoff points for parameters associated with the 90-day MRS score.	In different time windows, there are variations in inter-rater reliability among patients. Consistency among professionals is highest for moderate treatment times. The presence of collateral circulation is associated with favorable treatment outcomes, and the pre-intervention ASPECTS is a crucial predictor, especially when treatment initiation exceeds 200 minutes. The Tan score is also effective for ultra-acute strokes (OTI < 100 min).	Overall, there was good correlation without significant differences between the HVs and the FIVs with package A (r = 0.78, p < 0.001) being slightly superior to B and C. However, levels of agreement were very wide for all software applications in Bland-Altman analysis. In cases of large infarcts exceeding 150 mL the performance of the automated software solutions generally decreased. Subgroup analysis revealed the FIV to be generally underestimated in patients with HIR ≥ 0.6 (p < 0.05). In the subgroup with favorable HIR, however, there was a trend towards an overestimation of the FIV. Nevertheless, packages A and B showed good correlation between the HVs and FIVs without significant differences (p > 0.2), while only package C significantly overestimated the FIV (−54.6 ± 56.0 mL, p = 0.001). The rate of modified Rankin Scale (mRS) 0−3 after 3 months was significantly higher in favorable vs. unfavorable HIR (42.1% vs. 13.3%, p = 0.02). Lower HIR was associated with higher Alberta Stroke Program Early CT Score (ASPECTS) at presentation and on follow-up imaging, lower risk of malignant edema, and better outcome (p < 0.05).
**CONCLUSIONS**	Machine learning can be employed to predict the recovery outcomes of stroke patients. Initial imaging information is sufficient, and incorporating 24-hour information enhances accuracy. Considering reperfusion status aids in assessing treatment risks and benefits.	Imaging features from radiomics can serve as tools for predicting the risk of mMCAi.	CTA-venous-ASPECTS exhibits the highest area under the curve. This study indicates a nearly perfect correlation between CTA-venous-ASPECTS and the final infarct size, along with a significant association with the 90-day MRS score.	The sensitivity of NCCT in detecting rapid stroke progression decreases. In ASPECTS assessments based on both manual and machine learning approaches, the reliability and consistency of scores between acute and follow-up ASPECTS decrease during short-term OTIs. In cases of hyperacute stroke, the status of collateral circulation in CT angiography may contribute to improving the prediction of clinical outcomes and explaining the reasons for reperfusion failure.	HIR can serve as a valuable parameter for outcome prediction and aid in deciding whether to proceed with MT in delicate situations.
**PMID**	37287309	36934582	37437435	37581657	37607843
**YEAR**	2023	2023	2023	2023	2023
**LEARNING APPROACH**	machine learning	machine learning	machine learning	machine learning	machine learning
**PRIMARY AUTHOR**	Xiang S	Weng S	Zhang L	Brugnara G	Shen GC
**DISEASE**	Ischemic Stroke Patients with Large Vessel Occlusion beyond the Therapeutic Time Window	ischemic stroke	Acute ischemic stroke	acute ischemic stroke	estimated infarct core volume in the patients with acute ischaemic stroke
**DATA VOLUME**	54 patients were retrospectively divided into two groups based on the treatment methods: the mechanical thrombectomy group had 21 patients and the conservative treatment group had 33 patients	CTA and MRI images from 97 patients	Clinical data and NCCT (non-contrast computed tomography) images from 240 patients with acute ischemic stroke (AIS).	A total of 1103 consecutive patients, who underwent endovascular treatment (EVT) for occlusion in the territory of the middle cerebral artery, were included.	44 patients undergoing endovascular treatment.
**DATA TYPE**	CTA	CTA、DWI	Non-contrast computed tomography	native cranial computed tomography (NCCT)	mCTA
**METHODS**	machine learning model	machine learning model	Support vector machine	machine learning models	multi-scale three-dimensional convolutional neural network
**RESULTS**	In patients treated with mechanical thrombectomy guided by CTP imaging, the post-treatment NIHSS scores were significantly better than those in the conventional treatment group, with statistical significance (P < 0.005). In one case assessed using artificial intelligence-assisted CTP imaging, the infarct core volume was larger than that shown by DWI, while in the remaining 13 patients, the infarct core volume was smaller than that indicated by DWI.	On two independent test sets, the accuracy (ACC) for the cross-validated dataset using the Adboost method was 0.8519, while the ACC for the independent test set using the SRC method was 0.8125.	A total of 1454 texture features were extracted from NCCT images. In the test cohort, ROC analysis revealed that the radiomics model and the fusion model exhibited AUCs of 0.705 and 0.857, respectively. The fusion model demonstrated an accuracy of 84.8% and sensitivity of 93.8%.	**38% of patients exhibited favorable clinical outcomes, while 26% experienced adverse outcomes at 90 days. The predictive performance of the machine learning model is significant, with an AUC of 0.775.**	The area under the ROC curve is 0.874. The mean onset-to-door time (ODT) and door-to-puncture time (DPT) are 75.6 and 16.3 minutes, respectively, with a successful reperfusion rate of 17.7%. The optimal threshold for predicting performance is an estimated infarct core volume ≤40.3 ml based on mCTA.
**CONCLUSIONS**	Artificial intelligence-assisted CTP diagnosis can facilitate rapid assessments independent of radiologists, but it may pose challenges in determining infarct core volumes.	This machine learning approach can effectively explore and accurately quantify features related to stroke prognosis, including vascular structure and stroke location.	The model based on NCCT radiomics and machine learning has high predictive efficiency for the prognosis of AIS patients receiving conventional treatment, which can be used to assist early personalized clinical therapy	Cortical atrophy emerges as an independent predictor of clinical prognosis in patients with acute ischemic stroke. The machine learning model demonstrates exceptional performance when comprehensively considering both clinical and imaging parameters.	mCTA-estimated IC volume might be promising for predicting the prognosis, and assisting in making individualized treatment decision in patients with AIS

#### 2.4.2 The application of deep learning in stroke

In 2019, Hilbert et al. collected CTA data from 1,526 ischemic stroke patients. Various preprocessing techniques were applied to the images, including dimension reduction using Maximum Intensity Projections (MIPs) and rigid registration using Elastix software. Additionally, they developed a Structured Receptive Field Neural Network (RFNN) model and incorporated unsupervised pretraining in a stack denoising autoencoder (AE) experiment to learn the encoding part of the AE network. Machine learning models based on 20 radiographic biomarkers manually scored by experts from the MR CLEAN Registry core laboratory were constructed. Logistic regression (LR) models and random forest classifiers (RFC) were utilized and compared with standard Deep Learning (DL) models (ResNet). Three training methods were devised using four balanced groups for cross-validation in 1,301 patients. Gradient-weighted class activation mapping (Grad-CAM) was employed for visualization to elucidate the contribution of convolutional feature maps in the input space. Ultimately, two visualization models were developed for predicting mRS and mTICI outcomes. The Deep Learning models exhibited superior performance in the cross-validation folds of four functional outcomes, yielding an average AUC of 0.71, and achieved an average AUC of 0.65 across all reperfusion folds, surpassing models based on traditional radiographic biomarkers. The AUC values for LR and RFC methods were 0.68 and 0.66, respectively, for favorable functional outcomes. However, for reperfusion prediction, both LR and RFC yielded an AUC of 0.52. In conclusion, the RFNN-ResNet model achieved the highest average AUC without pretraining with emission, while RFNN-ResNet-AE fine-tuning excelled in mTICI prediction. Their Deep Learning methods outperformed traditional approaches and can predict stroke outcomes without necessitating image annotation, offering faster processing speed. By enhancing the model, interpretability of the predictions was improved (50).

In 2021, Hokkinen et al., included data from 117 suspected stroke patients with CTA and follow-up data after admission. Preprocessing was done using 3D Slicer images and a trained and validated 3D CNN, evaluating the accuracy of outputs for two clinical time windows (0–6 and 6–24 h). The accuracy of CNN was assessed through visual evaluation of ASPECTS anatomical regions, validating the matching accuracy of the CNN in lesion location and final infarct location, compared with ischemic changes marked by radiologists. Finally, the performance of CNN and CTP-RAPID in determining eligibility for Endovascular Treatment (EVT) was compared, considering factors such as ischemic core volume and patient age. Using a manually fitted linear model, the research team assessed the segmented volume output derived from CNN and CTP-RAPID ischemic core volume for predicting final infarct volume. Pearson correlation coefficients were used to assess the correlation between them, and Bland–Altman plots were used to show the agreement between estimated infarct volume and final infarct volume, as well as the volume derivation between CNN and CTP-RAPID. The results showed that in the early 0–6 h time window, CNN had a correlation of *r* = 0.43 (*p* = 0.002) with final infarct volume, while CTP-RAPID had a correlation of *r* = 0.58 (*p* < 0.001). In the late 6–24 h time window, both CNN (*r* = 0.67, slope 1.2, *p* < 0.001) and CTP-RAPID (*r* = 0.82, slope 1.4, *p* < 0.001) showed significantly increased correlation. Compared to CTP-RAPID, CNN had a sensitivity of 0.38 and specificity of 0.89. The study suggests that CTA-based CNN, in patients successfully receiving EVT treatment, can detect anterior circulation ischemic stroke in the late time window (6–24 h) and has a moderate correlation with final infarct volume (51).

In 2021, Hokkinen et al. included 83 patients who received thrombolytic treatment or supportive care for CTA. They saved the images to a server and performed precise segmentation of the infarct area using the 3DSlicer image processing and visualization platform. The accuracy of the lesion location predicted by CNN was evaluated in comparison with the ASPECTS anatomical regions, and a detailed comparison was made with the CTP-RAPID software. By calculating a linear regression model and Pearson correlation coefficient (*r*) between the two, the results showed that the sensitivity of the CNN output was 0.71, specificity was 0.87, and accuracy was 0.80. For patients who did not receive thrombolytic treatment, there was excellent correlation between the final infarct volume and the estimated values from CNN output and CTP-RAPID, with correlation coefficients of *r* = 0.89 (95% CI 0.80–0.95) and *r* = 0.92 (95% CI 0.83–0.97), respectively. There was also a good correlation between the CNN output and CTP-RAPID ischemic core volume (*r* = 0.89, 95% CI 0.82–0.94). The conclusion of the study is that CTA-based CNN software demonstrates good estimation capabilities for infarct core volume in follow-up imaging studies, and its output exhibits significant correlation with CTP-RAPID ischemic core volume (52).

In 2021, Hakim et al. summarized the results of the ISLES 2018 challenge, which was participated in by 24 teams, and which included CTP and DWI images of 103 patients with acute large artery occlusion and anterior circulation ischemic stroke. Of these 103 patients were divided into two groups, 40 for the lesion-free test set and 63 for the training set. The data consisted of (1) CTP source data; (2) perfusion maps post-processed using the standard thresholding method (RAPID), i.e., cerebral blood flow (CBF), cerebral blood volume, mean passage time, and time to peak; (3) DWI lesion segmentation in a binary form; and (4) the DWI images themselves. Teams used different thresholds to calculate the mean and standard deviation of the Dice similarity coefficient (DSC), the mean absolute volume difference (VD), the accuracy and recall, including the Dice score, the Hausdorff distance (HD), the mean and absolute lesion VD, the accuracy, the recall, and the mean symmetry plane distance. Comparisons of non-normally distributed data, including comparisons of HD and mean symmetric surface distance, were performed using the Wilcoxon signed rank test to identify the best performing cases for each team. Results showed that among the best performing cases, the median DWI capacity was 7.2 (IQR) and the median absolute VD was 26.41. The study conclusions suggest that CTP-based machine learning methods can more accurately predict infarcted tissue (53).

In 2022, Ramos et al. conducted a comprehensive analysis of CTA data from 3,279 patients who underwent acute ischemic stroke EVT. They utilized two training model approaches, one based on radiomics and another combining imaging with clinical information. After preprocessing the data, 1,260 features were computed in 70 regions of the brain map, which were then reduced to 68 features. Training was carried out using the ResNet10 architecture for up to 75 epochs, incorporating transfer learning with an additional 50 epochs, and enhancing weights by adding SE modules before the fully connected layer. The results revealed that 37% of patients exhibited modified Rankin Scale (mRS) ≤ 2 in terms of favorable functional outcomes, while 60% achieved improved Thrombolysis in Cerebral Infarction (eTICI) ≥ 2b in terms of reperfusion. At 90 days, 37% of patients had good functional outcomes, and 60% showed favorable reperfusion after treatment. In predicting functional outcomes, the radiomics method performed the best in clinical experiments, achieving an AUC of 0.81. The study suggests that a single Deep Learning method (ResNet10) performed relatively poorly in predicting favorable functional outcomes. The combined approach of clinical and radiomics data demonstrated good performance in predicting patient functional outcomes (54).

In 2022, Winder et al. conducted a study involving 145 patients with acute ischemic stroke who underwent ERASER thrombectomy or other treatments. The research utilized AnToNIa software for perfusion imaging analysis, processing CT perfusion maps (CTC), mean square deviation, and baseline average images. Block-cyclic singular value decomposition, truncation threshold at 15%, and automatically calculated arterial input function were employed for deconvolution to generate residue curves (RC), thereby creating perfusion parameter maps for CBF, cerebral blood volume (CBV), mean transit time (MTT), and time to peak (Tmax). Additionally, brain tissue masks were generated. NCCT images underwent segmentation using AnToNIa and ITK-SNAP software tools, and registration to the baseline average image was performed using the SimpleITK and ANTs software packages. In the machine learning phase, each dataset underwent masking of the ipsilateral hemisphere, and CTC and RC data were cropped to 32 time points of interest, followed by corresponding processing. The tissue outcome prediction phase included model training, model testing, binarization, and statistical evaluation steps. Using Deep Learning models, image analysis was performed on 222 patients from the I-KNOW multicenter and remote ischemic preprocessing, training with TensorFlow and Python. The study evaluated the radiological outcomes of a subset of patients receiving intravenous rtPA and compared the performance of different models. The results showed that, compared to other models, CNNdeep performed better with an AUC of 0.88 ± 0.12, demonstrating significant differences from GLM, CNNTmax, and ADCthres. Overall, using multiple biomarkers as inputs in Deep Learning models achieved better predictive performance (55).

In 2022, Jabal et al. included 443 patients with AIS who underwent thrombectomy. Quantitative imaging features were extracted from clinical information and CT images using the e-Stroke software. The features were categorized into four classes, and additional new features were extracted. Machine learning (ML) algorithms, including k-Nearest Neighbors, Random Forest (RF), Gradient Boosting (GB), and Extreme Gradient Boosting (XGBoost), were constructed using the Scikit-learn library. The algorithms were optimized through the Optuna framework to differentiate and segment ASPECTS and output the total volume, volume for each ASPECTS region, and total e-ASPECTS volume. Simultaneously, the e-CTA software was used to identify the location of large vessel occlusion and quantify the volume percentage of collateral circulation defects to the total volume, as well as the absolute volume of vascular density defects in the MCA region relative to the contralateral hemisphere. Results showed that 101 patients had a favorable functional outcome (mRS-90 ≤ 2), while 192 patients had an unfavorable functional outcome (mRS-90 > 2). Non-enhanced CT imaging features associated with a favorable outcome included larger e-ASPECTS, larger brain volume, smaller cortical cerebrospinal fluid volume, smaller lateral ventricle volume, smaller acute ischemic volume, and smaller non-acute ischemic volume. Regarding imaging features, the XGBoost model performed the best with an AUC of 79%. Considering both clinical and radiological features, XGBoost remained the optimal model with an AUC of 80%. After Bayesian hyperparameter tuning and 10-fold stratified cross-validation, the optimized XGBoost model demonstrated a final performance on the patient test set with an AUC of 84%, accuracy of 77%, F1 score (mRS ≤ 2) of 67%, and F1 score (mRS > 2) of 82% (56).

In 2022, Amador et al. conducted a study on acute ischemic stroke, retrospectively collecting baseline CTP images from 147 patients. They preprocessed the images using the AnToNIa perfusion analysis software, which included motion correction, baseline correction, time smoothing, and interpolation. Building upon the preprocessing, they employed a Deep Learning approach to automatically identify the arterial input function (AIF). This involved architectures such as U-Net and temporal convolutional networks, directly utilizing the raw 4D CTP images for spatiotemporal analysis to predict treatment-dependent lesion outcomes in AIS patients. The study employed a 10-fold cross-validation scheme and, based on follow-up lesion volumes, trained, and evaluated the proposed Deep Learning method alongside a Tmax thresholding approach. All Deep Learning models underwent training for 100 epochs, and three performance evaluation metrics proposed by Winzeck et al. (57) were used for analysis. The results indicated that the 3D + time model performed the best in predicting stroke lesions, with a DSC of 0.30, a HD of 9.5 mm, and a volume error of 3.0 mL. In contrast, the performance of the Tmax thresholding method was the poorest, with a DSC of 0.24, HD of 14.4 mm, and volume error of 86.8 mL. The 2D + time model and the baseline method exhibited slightly lower average performance but were still considered acceptable, with an average volume error of 21.9 mL (58).

In 2023, Wouters et al. included 228 acute ischemic stroke patients with 127 in the training set and 101 in the validation set. They utilized CTP data from the MRCLEAN trial-derived cohort for training a DL model, and internal validation was performed after integrating clinical data. External validation used an independent dataset from the CRISP study. The study compared the performance of the DL model with the RAPID software, which uses deconvolution/thresholding methods, in predicting final infarct volume. Additionally, analyses of patient reperfusion grades, lesion growth rates, and relevant statistical analyses were conducted. The results showed that, in the analysis of 108 patients based on baseline CTP and actual infarct volume using RAPID, the DL model outperformed the RAPID software, with a mean absolute difference (MAD) of 34.5 mL (SD 29.4), compared to RAPID software’s MAD of 52.4 mL (SD 49.8) (*p* < 0.01). For the 19 patients with intermediate reperfusion in the MR CLEAN study, the DL model had a MAD of 36.7 mL (SD 38.3), with no significant difference compared to the fully or non-reperfused groups (*p* = 0.64). ROC curve analysis indicated an optimal threshold for infarct growth at 0.36, with a median growth rate of 2.7 mL/h in patients with HIR < 0.36 and 8.5 mL/h in patients with HIR ≥ 0.36 (*p* < 0.01) (59).

The main information of the above included literatures is shown in Table 2.

**Table 2:** Summary of papers on deep learning for CT on rehabilitation of Ischemic stroke.

PMID	31707199	34868662	34164743	33957774	35432171
YEAR	2019	2021	2021	2021	2022
LEARNING APPROACH	deep learning	deep learning	deep learning	deep learning	deep learning
PRIMARY AUTHOR	Hilbert A	Hokkinen L	Hokkinen L	Hakim A	Ramos LA
DISEASE	Acute Ischemic Stroke	anterior circulation ischemic stroke	anterior cerebral circulation ischaemic stroke	Acute Ischemia	Acute Ischemic Stroke
DATA VOLUME	the MR CLEAN Registry dataset with 1301 patients	117 suspected stroke patients.	83 consecutive stroke cases undergoing thrombolytic therapy or alternative treatments	Among 103 patients with acute anterior circulation ischemic stroke due to large vessel occlusion, 40 constituted the lesion-free test set, while the remaining 63 formed the training group.	3279patients from the MR CLEAN Registry
DATA TYPE	Clinical variables and radiological image biomarkers (including age, pre-stroke mRS, NIHSS, occlusion location, ASPECTS, etc.)	CTA	CTA	CTP、DWI	radiomics features、 images and the clinical data
METHODS	Residual Neural Network (ResNet)	Convolutional neural network(CNN)	convolutional neural network	Machine learning method	3D deep learning models、machine learning models
RESULTS	Deep learning models demonstrated superior performance in functional outcome (average AUC of 0.71) and reperfusion (average AUC of 0.65).	The final infarct volume correlation for the Convolutional Neural Network (CNN) was r=0.43, while for CTP-RAPID, it was r=0.58. Within the 6-24 hour time window, both CNN (r=0.67, slope 1.2) and CTP-RAPID (r=0.82, slope 1.4) showed a significantly increased correlation. Compared to CTP-RAPID, CNN demonstrated a sensitivity of 0.38 and specificity of 0.89.	The sensitivity, specificity, and accuracy of the CNN stand at 0.71, 0.87, and 0.80, respectively. The correlation coefficient with manual segmentation is 0.83. For patients not subjected to thrombolytic therapy, noteworthy correlations emerge between the CNN output and CTP-RAPID estimated values, with correlation coefficients of r=0.89 and r=0.92, respectively. Additionally, a robust correlation of r=0.89 is observed between the CNN output and CTP-RAPID ischemic core volume.	In the best-performing cases, the median DWI volume was 7.2 (IQR), with a median absolute vascular density (VD) of 26.41	Combining image data with clinical data did not yield a significant improvement in mRS prediction (mean AUC of 0.81 vs. 0.80) compared to using clinical data alone, irrespective of the approach. However, for predicting reperfusion, a significant enhancement was observed with the combination of image and clinical features (mean AUC of 0.54 vs. 0.61), with the deep learning approach achieving the highest AUC.
CONCLUSIONS	n our dataset, automated image analysis using deep learning methods demonstrates superior performance in predicting stroke outcomes, with the potential to enhance treatment selection.	A CTA-based CNN method had moderate correlation with final infarct volumes in the late time window in patients successfully treated with EVT.	The CNN software based on CTA can provide robust estimates of infarct core volume. The infarct volume derived from CNN exhibits a strong correlation with the ischemic core volume from CTP-RAPID.	Machine learning methods may predict infarcted tissue from CTP with improved accuracy compared with threshold-based methods used in clinical routine.	The integration of radiomics and deep learning image features with clinical data significantly improves the prediction of favorable reperfusion.
PMID	36408399	35665041	36103772	34587794	
YEAR	2022	2022	2022	2023	
LEARNING APPROACH	deep learning	deep learning	deep learning	deep learning	
PRIMARY AUTHOR	Winder AJ	Jabal MS	Amador K	Wouters	
DISEASE	acute ischemic stroke	estimated infarct core volume in the patients with acute ischaemic stroke	spatio-temporal convolutional neural networks	Acute Ischemic Stroke	
DATA VOLUME	145 patients with acute ischemic stroke who underwent intravenous thrombolysis treatment, following the conventions of scientific literature	293 patients undergoing thrombectomy.	147 patients from the multicenter, prospective ERASER study	228 acute ischemic stroke patients with 127 in the training set and 101 in the validation set.	
DATA TYPE	CT	CT/CTA	CTP	CTP	
METHODS	DCN	multi-scale three-dimensional convolutional neural network	4D CT perfusion imaging using spatio-temporal convolutional neural networks	DL model with the RAPID software	
RESULTS	DCN: 0.287, RDF: 0.262, Tmax-thresholding: 0.249, deconvolved residual curves: 0.286, source concentration-time curves: 0.296	In a cohort of 101 patients, favorable outcomes (mRS-90 ≤ 2) were observed, while 192 patients experienced unfavorable outcomes (mRS-90 > 2). The XGBoost model demonstrated optimal performance when considering both imaging features and clinical considerations, achieving AUCs of 79% and 80%, respectively. Following optimization, the final performance of the XGBoost model on the patient test set was characterized by an AUC of 84%, accuracy of 77%, F1 score (mRS ≤ 2) of 67%, and F1 score (mRS > 2) of 82%.	The 3D+time model demonstrates optimal performance, boasting a Dice Similarity Coefficient (DSC) of 0.30, Hausdorff Distance (HD) of 9.5 mm, and a volume error of 3.0 mL. In contrast, the Tmax threshold method exhibits the least favorable performance, with a DSC of 0.24, HD of 14.4 mm, and a volume error of 86.8 mL. The 2D+time model, while displaying slightly lower average performance, still maintains acceptability, with an average volume error of 21.9 mL.	The results showed that, in the analysis of 108 patients based on baseline CTP and actual infarct volume using RAPID, the DL model outperformed the RAPID software, with a mean absolute difference (MAD) of 34.5 ml (SD 29.4), compared to RAPID software's MAD of 52.4 ml (SD 49.8) (p < 0.01). For the 19 patients with intermediate reperfusion in the MR CLEAN study, the DL model had a MAD of 36.7 ml (SD 38.3), with no significant difference compared to the fully or non-reperfused groups (p = 0.64).	
CONCLUSIONS	Through DCN, utilizing features optimized from source concentration-time curves, the best predictions for tissue outcomes are provided.	The value of machine learning lies in integrating essential clinical information and automated imaging features for predicting functional outcomes three months after mechanical thrombectomy.	4D CTP datasets include more predictive information than perfusion parameter maps, and that the proposed method is an efficient approach to make use of this complex data	ROC curve analysis indicated an optimal threshold for infarct growth at 0.36,	

### 2.5 Artificial intelligence-based prediction of stroke prognosis using MRI

MRI has various imaging sequences, such as diffusion-weighted MRI, perfusion MRI, T2 sequences, etc., which can assess different structural and functional features of brain tissue. Diffusion MRI can detect cytotoxic edema, which is the most sensitive core indicator of ischemic stroke. In the region of cytotoxic edema, water molecules move from extracellular space to intracellular space, and diffusion is restricted. Diffusion-weighted imaging can detect ischemic injury within minutes after the onset of ischemic stroke, showing a significant high signal (60). Studies have indicated that the sensitivity of MRI in diagnosing acute ischemic stroke is 83% (61). Traditional MRI sequences, such as T1, T2, and FLAIR, become sensitive to ischemic changes after a net increase in brain tissue water content, allowing detection of ischemic changes hours after symptom onset (62). Perfusion-weighted imaging (PWI) can provide an assessment of cerebral blood flow perfusion. By combining information from PWI and DWI sequences, the size and location of the ischemic penumbra can be evaluated.

MRI vascular imaging is a powerful tool for detecting vascular stenosis or occlusion, but it is more time-consuming than CTA and may not be available around the clock or in emergencies at all hospitals. Despite its limitations, MRA has unique advantages in diagnosing stenosis, occlusion, etc. In most studies, MRA has a sensitivity and specificity close to 100% for detecting carotid artery occlusion (63). If MRA shows no stenosis or stenosis less than 70%, further diagnostic evaluation is usually not necessary.

**Figure 5:**
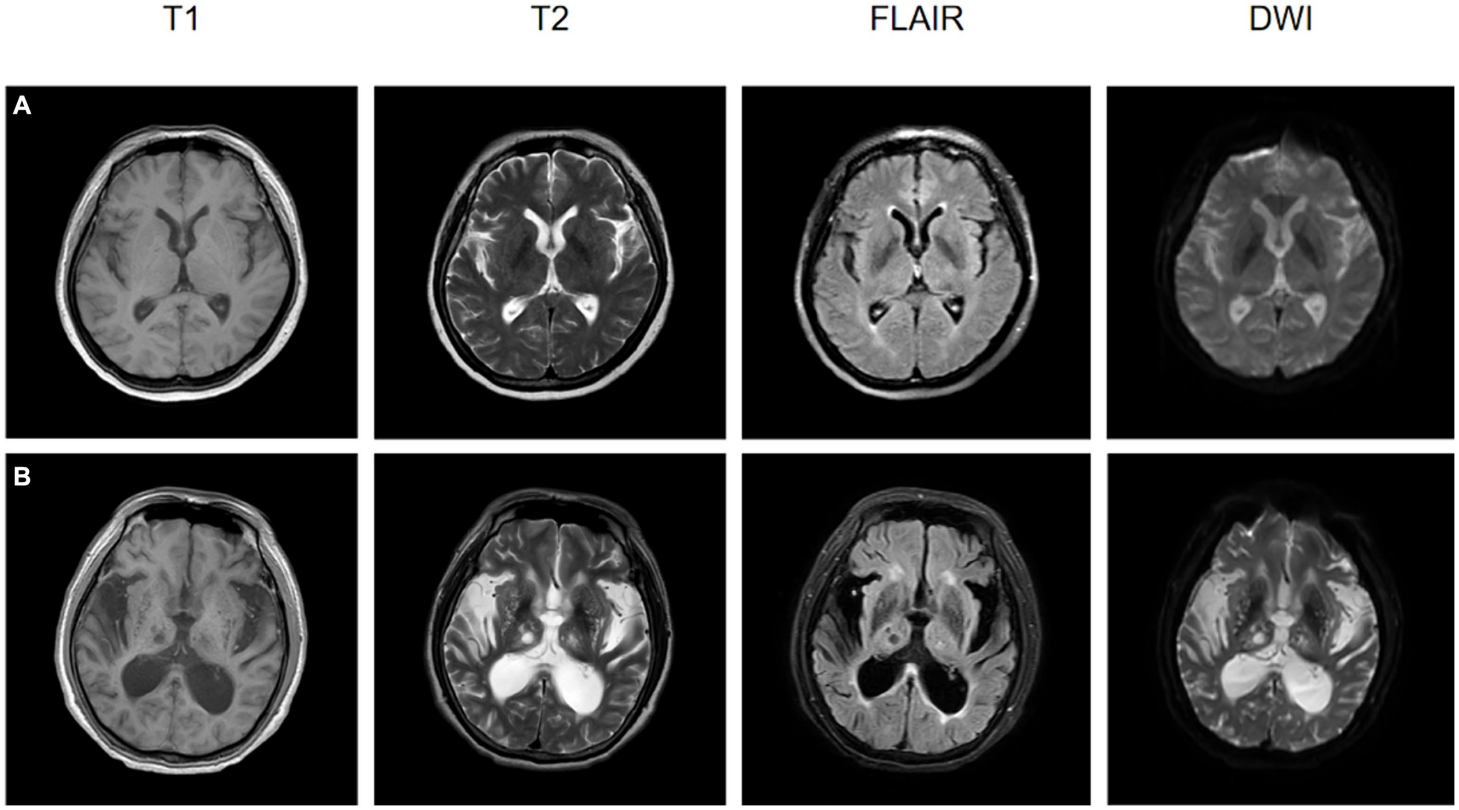
The MRI images at the onset of ischemic stroke for two patients, both of whom underwent rehabilitation training after their conditions stabilized, are presented. **(A)** A 57-year-old female patient, post-rehabilitation Berg Balance Scale score of 2, Functional Independence Measure (FIM) score of 6, and Modified Barthel Index of 3. **(B)** A 72-year-old male patient, post-rehabilitation Berg Balance Scale score of 0, FIM score of 1, and Modified Barthel Index of 0.

MRI has been used as a preferred modality for the treatment and secondary prevention of acute ischemic stroke, as show in Figure 5. While CT is most commonly employed for assessing acute stroke patients, the high signal-to-noise ratio and the ability to identify damaged brain tissue make MRI a crucial imaging modality for stroke diagnosis, prognosis, and prevention. Evaluation of ischemic stroke typically involves multiple imaging parameters, with changes in cerebral blood flow being a primary pathological alteration in ischemic stroke. MRI sequences, such as dynamic contrast-enhanced perfusion, can estimate the penumbral tissue, which is at risk of infarction without reperfusion treatment but has not yet undergone irreversible damage. Additionally, it can assess cerebral vascular reserve (CVR) to better select cerebrovascular intervention measures. CVR is defined as the ability to increase CBF in response to vascular dilation stimuli. In patients with reduced CVR, there is often an increased risk of stroke when CVR is diminished, especially in those with chronic cerebrovascular diseases. Arterial spin labeling (ASL) is an enhanced MRI sequence used to measure CBF, offering a promising technique for evaluating acute ischemic stroke and potentially identifying patients at higher risk of future strokes (64).

#### 2.5.1 The application of machine learning in stroke

In 2005, Gottup et al. collected MRI data from 14 patients with acute stroke. The performance was measured using AUC. Three different implementations of the instance-based method—k-NN, Gaussian weighted, and constant radius search classification—were applied for data analysis. The results indicated that the performance of the optimal k-NN and Gaussian weighted algorithms did not shown a significant difference, but both were markedly superior to the constant radius implementation. Through a qualitative analysis of the distribution of instances in the feature space, it was observed that non-infarct instances tended to cluster together, while infarct instances were more dispersed in the feature space. Additionally, the analysis suggested the existence of feature space regions occupied exclusively by infarct instances, which were not present for non-infarct instances (65).

In 2015, Kim et al. enrolled 35 ischemic stroke patients with visual field defects (VFD) caused by posterior cerebral artery (PCA) infarction. All these patients underwent MRI scans. After transforming the lesion locations into standard maps, the ischemic lesion area range (rEILs) for each cortical area was measured. Significant improvement in VFD was defined as a provisional improvement of 20% 3 months after the stroke. The performance of clinical and radiological variables in predicting significant improvement was measured using support vector machines. Clinical variables, baseline visual field scores, lesion volumes, and rEIL were compared between the significantly improved and less improved groups. Support vector machines with a linear kernel were employed to train and validate the prognostic classifier. The results showed that left PCA infarction, pre-stroke MRI time, and rEIL in the tongue, corpus callosum, and cuneal cortex were good prognostic indicators for lateralized VFD. Compared to clinical variables, the combination of rEIL in various cortical subregions demonstrated a better predictive effect on lateralized VFD. Adding rEIL to other variables improved the prognostic prediction of lateralized VFD (66).

In 2020, Grosse et al. included 99 patients with acute ischemic stroke for multi-parametric MRI data. They used the AnToNIa software tool for apparent diffusion coefficient (ADC) map calculation, intraslice motion correction of PWI sequences, and atlas-based methods for automatic extraction of arterial input functions, among other preprocessing steps. Perfusion parameter maps were computed using a block-circulant singular value decomposition method, and the FLAIR dataset was segmented. Image registration to the Montreal Neurological Institute (MNI) brain atlas was performed, and a combination of LR and RF algorithms was used for mixed tissue outcome prediction. Twenty-one prediction models were evaluated using Receiver Operating Characteristic Area Under the Curve (ROC AUC), Dice similarity index, sensitivity, and specificity. Single-sided paired *t*-tests were employed to assess Dice and ROC AUC values, and the average and median of local LR model coefficients were calculated for each MNI structural brain region. The study results demonstrated that the mixed LR model performed best in terms of the average ROC AUC value (0.872 ± 0.092), while the mixed RF model was optimal for the average Dice coefficient (0.353 ± 0.220). The mixed LR model showed the highest average values for ROC AUC and Dice coefficient, followed by the mixed RF model. The mixed model significantly improved the effect size at the 0.01 level, including ROC AUC and Dice values. The mixed LR model had the highest average values for ROC AUC, Dice coefficient, sensitivity, and specificity, followed by local LR, mixed, and local RF models, as well as global LR and RF models (67).

In 2021, Hamann et al. included clinical and imaging data from a cohort of 222 patients who underwent EVT for acute ischemic stroke caused by middle cerebral artery (MCA)-M1 occlusion at the Bern University Hospital between January 2012 and August 2017. The data used for predictive analysis was limited to diffusion-weighted and perfusion images. Imaging data underwent post-processing using the Acute Stroke Care plugin of Olea Sphere. A predictive model for favorable functional outcomes was developed using clinical variables and magnetic resonance imaging features based on regions of interest (ROIs). The study assessed the predictive capability of different patient characteristics and imaging variables, both individually and in combination, and evaluated overall performance based on the AUC values and Brier scores for the entire test set. The results indicated a successful revascularization rate of 78%, with 54% of patients achieving a favorable outcome (modified Rankin Scale score 0–2). Small infarct size was associated with a favorable functional outcome, while older age was related to a reduced chance of favorable outcomes and functional improvement. The use of isolated imaging information as a predictor for functional outcomes showed relatively poor performance. No significant differences were observed between the predictive variable sets when imaging variables were added to patient characteristics (68).

In 2022, Graaf et al. analyzed patients who had successful reperfusion in the MR CLEAN registry center from March 2014 to November 2017. Initially, they constructed a multivariable ordinal regression model to predict functional outcomes measured by the mRS at 90 days. Four groups of predictive factors were included: baseline patient factors, imaging factors, treatment factors, and postoperative factors (i.e., adverse events). Each group of predictive factors was incrementally added to the basic model, which only included baseline patient factors, and the overall explained variance of the most comprehensive model was subsequently evaluated. The results indicated that the most important predictive factors for mRS were baseline patient factors and postoperative factors. Among patients with successful reperfusion, the five most important individual predictive factors for functional outcomes at 90 days were pre-stroke mRS, baseline NIHSS, symptomatic intracranial hemorrhage (sICH), age, and pneumonia. Stroke patients with sICH had a 54% lower probability of functional independence compared to those without sICH, and patients with pneumonia had a 21% lower probability of functional independence than those without pneumonia. This study suggests that both patient and postoperative factors are crucial predictors of successful reperfusion outcomes in ischemic stroke patients (69).

In 2021, Abedi et al. developed classification models for six prediction windows by incorporating MRI data from 7,144 patients with acute ischemic stroke. Three algorithms, LR, XGB, and RF, were employed in the study. The research data were randomly split into 80: 20 training and testing sets, using RF and LR as baseline metrics. Ten repetitions of 5-fold cross-validation (CV) training were performed. Among the 7,144 patients meeting inclusion criteria, 5,347 did not experience a stroke after 2 years, 605 died within 1 month, 1,380 died within 1 year, and 1,797 died within 2 years. On the test dataset, the average Area Under the Receiver Operating Characteristic curve (AUROC) ranged from 0.76 to 0.81. The RF-based model performed best in the 1-month window (AUROC = 0.82), with the highest Negative Predictive Value (NPV) of 91.1 for shorter prediction windows. The RF model achieved the highest PPV at the 6-month window (0.92), while the XGB-based model had the highest accuracy (precision of 0.89) in the 1-month window. Age, hemoglobin levels, and BMI were identified as the top three relevant factors across different prediction windows, with average overall importance scores of 96.3, 68.2, and 55.5%, respectively (70).

In 2022, Elsaid et al. recruited 354 patients using a systematic random technique (every three admissions) from the Stroke and Intensive Care Unit (ICU) at Zagazig University Hospital in Egypt. The included data comprised routine MR and diffusion-weighted images for each patient. The team optimized several machines learning algorithms, including LRC, SVC, RFC, GBC, and MLPC. They evaluated the predictive performance of the models using ROC and explored the interactions among predictive factors using Generalized Additive Models (GAM). The results indicated a 19.8% occurrence rate of HT in ischemic stroke patients. Infarct size, cerebral microbleeds (CMB), and NIHSS were identified as the best predictors for HT. RFC and GBC outperformed LRC and MLPC significantly. SVC performed better than LRC and MLPC but lacked statistical significance. There was no significant difference between LRC and MLPC (71).

In 2022, Guo et al. conducted a comprehensive analysis by including 156 patients with 88 DSC-PWI images. They preprocessed the DSC-PWI data and assessed the role of Dynamic R2* (DRF) in the diagnosis and prognosis prediction of ischemic stroke patients. The study segmented DSC-PWI images into N 3D images and calculated the performance of brain tissue DRF through four combination methods. Ten machine learning models, including SVM, Decision Tree (DT), Adaboost Classifier (Ada), Neural Network (NN), and others, were used for performance evaluation based on AUC, with AUC calculated using 10-fold cross-validation. Analysis of 78 DSC-PWI images detected 50 cases of ischemic stroke, including 60 patients with NIHSS scores of 95 and 66 patients with mRS scores less than 2 at 101 days. In terms of feature extraction, which included First_order, GLCM, GLDM, GLRLM, GLSZM, NGTDM, etc., the *p* values ranged from 0.0123 ± 0.0144. The *p* value for DRF significantly correlated with NIHSS assessment was 8324.0 ± 0232.0, totaling 156. For outcome prediction, 144 significant DRFs were extracted with a *p* value of 9203.0 ± 0238.0, including First_order, GLCM, GLDM, GLRRM, GLSZM, NGTDM, etc. Reduction in dimensions through PCA, ICA, t-SNE, IOSMAP, and UMAP methods resulted in *R* values of 0.110 ± 0.121, 0.140 ± 0.079, 0.110 ± 0.121, 0.294 ± 0.139, and 0.098 ± 0.133, respectively. The conclusion states that the study results indicate that different feature extraction and dimensionality reduction methods can achieve better performance in the detection, assessment, and outcome prediction of ischemic stroke. In some cases, features selected by Lasso demonstrated superior performance, increasing AUC for stroke detection by 19.4% (from 0.731 to 0.925), NIHSS assessment by 20.1% (from 0.652 to 0.853), and prognosis prediction by 14.9% (from 0.679 to 0.828) (72).

In 2022, Li et al., conducted a study involving 260 patients with acute ischemic stroke, incorporating a total of 620 DWI images. Initially, neuroradiologists selected ROIs, followed by ROI segmentation and normalization preprocessing. They integrated a SVM algorithm with a Least Absolute Shrinkage and Selection Operator (LASSO) regression model and optimized model parameters through 10-fold cross-validation. The predictive performance of the machine learning model was assessed using AUC of ROC curve. The study results revealed that among the 260 patients with acute ischemic stroke, there were 109 and 46 cases of favorable outcomes in the training and test sets, respectively. The LASSO regression model identified four features, with the highest-weighted coefficient attributed to “sqyareriit-IV.” ROC curve analysis demonstrated that in the training set, the AUC for predicting the prognosis after mechanical thrombectomy was 0.945 (95% CI: 0.890–0.975), and in the test set, it was 0.920 (95% CI, 0.849–0.981) (73).

In 2023, Luo et al. conducted a study analyzing 132 patients with Basilar Artery Occlusion (ABAO), randomly dividing them into a training group (*n* = 106) and a test group (*n* = 26). They extracted 1,130 radiomic features from DWI images and employed the Least Absolute Shrinkage and Selection Operator (LASSO) regression method for feature selection. Radiomic and clinical models were constructed using SVM, and the models were evaluated using ROC and decision curves. The results indicated that AUC of the ROC curve for the radiomic-clinical model was 0.897 in the training group and 0.935 in the test group. The AUC for the radiomic model was 0.887 in the training group and 0.840 in the test group. The AUC for the clinical model was 0.746 in the training group and 0.766 in the test group. Importantly, the AUC of the radiomic-clinical model was significantly greater than that of the clinical model (74).

In 2023, Xu et al. included 314 patients with acute corpus callosum infarction (CC). Basic clinical and radiological information was obtained through the Electronic Medical Records (EMR) management system. Neuroimaging evidence was collected from MRI, MRA, or CTA. For patients diagnosed with CC infarction 1 year after onset, the team used the Behavioral Risk Factor Surveillance System (BRFSS) questionnaire to identify subjective cognitive decline (SCD). The team established seven machine learning models, including XGBoost, LR, LightGBM, AdaBoost, GNB, CNB, and SVM. They compared the predictive performance of these models using different metrics. The results indicated that the LR model outperformed the other six machine learning models in predicting SCD after CC infarction. Through LASSO and SHAP analyses, the team identified the top nine important predictive factors from the LR model output. Additionally, they discovered factors independently associated with cognitive outcomes (75).

In 2023, Wang et al. conducted a study involving 2015 patients who experienced ischemic strokes within 650 h. The study utilized MRI images and follow-up data. NeuBrainCARE software was employed to manually measure the infarct volume, represented by the ADC. Additionally, two radiologists assessed the burden of small vessel disease (SVD). The researchers employed a bidirectional stepwise regression method to select indicators in the LR model and applied three machine learning algorithms (Gaussian process regression, random forest, and extreme gradient boosting) to establish predictive models. The results indicated that the LR model (SVO-AIS) achieved an AUC of 0.86 [0.78–0.94] for favorable outcomes and an AUC of 0.88 [0.8–0.96] for good outcomes. The LR model (LAA-AIS) had AUC values of 0.73 [0.54–0.91] for favorable outcomes and 0.75 [0.59–0.91] for good outcomes. The GPR model (SVO-AIS) achieved an AUC of 0.86 [0.77–0.95] for favorable outcomes and an AUC of 0.86 [0.77–0.96] for good outcomes. The GPR model (LAA-AIS) had AUC values of 0.65 [0.47–0.83] for favorable outcomes and 0.66 [0.49–0.84] for good outcomes. The GOA-RF model (SVO-AIS) demonstrated an AUC of 0.85 [0.75–0.94] for favorable outcomes and 0.84 [0.74–0.94] for good outcomes. The GOA-RF model (LAA-AIS) achieved AUC values of 0.66 [0.49–0.84] for favorable outcomes and 0.68 [0.51–0.86] for good outcomes. The GOA-XGBoost model exhibited AUC values of 0.87 [0.79–0.96] for favorable outcomes and 0.85 [0.76–0.94] for good outcomes. In the LAA-AIS population, the AUC values were 0.91 [0.84–0.97] for favorable outcomes and 0.90 [0.83–0.97] for good outcomes (76).

In 2023, Yu et al. investigated the demographic characteristics, clinical data, and MR data of 180 patients with AIS. They manually delineated ROI for acute ischemic lesions on DWI images using MRIcron software. The data from all modalities, including ADC, FLAIR, SWI, and T1-2w, were aligned with the DWI images using SPM1 software. Radiomic analysis was performed on the MRI data of the five modalities, extracting a total of 946 features per image. Additionally, 14 shape features of the lesion regions were extracted. The scikit-learn package was utilized for feature selection using the recursive feature elimination (RFE) method. Ultimately, 16 image features were selected for training machine learning models, including SVM, RF, LightGBM, CatBoost, and XGBoost. Statistical analysis was conducted using SPSS 21.0. The results showed that out of 148 patients, 83 (56.1%) had a favorable prognosis, while 65 (43.9%) had an unfavorable prognosis. For each MRI modality, three optimal radiomic features (DWI, ADC, FLAIR, SWI, and T1w) and one optimal feature related to lesion shape were selected as key features in the machine learning models. The accuracy of the models was as follows: SVM model, 79%; RF model, 82%; LightGBM model, 83%; CatBoost model, 81%; and XGBoost model, 80%. This study revealed the potential value of a multimodal radiomic approach in accurately predicting the clinical outcomes of patients with AIS (77).

In 2023, Lee et al. included 3,687 patients with acute ischemic stroke, along with their MRI and relevant clinical information. The Rankin scale, assessed by neurologists, was used for outcome evaluation. The team employed multiple imputation by chained equations (MICE) to handle outliers and missing data and normalized the data using MinMaxScaler. They developed models based on three algorithms: random forest, XGBoost, and LGBM. The TreeSHAP method was used to calculate interaction effects between features. Model performance was evaluated using AUC-ROC, revealing that 30.4% of patients had unfavorable outcomes, with external validation data showing rates of 37.2 and 29.1%. The AUROC for the internal test set, external validation sets A and B were 0.790, 0.791, and 0.873, respectively, while the Brier scores were 0.172, 0.202, and 0.141. In summary, the models demonstrated overall good performance (78).

In 2023, Weng et al. included 97 stroke patients with CTA and MRI images. Preprocessing of CTA and DWI images was conducted in MATLAB 2019. For CTA images, the processing involved feature extraction for 116 brain regions, image registration, segmentation of cerebral vessels using the Unet model, and calculation of vascular volume and length features. For DWI image processing, it included location feature extraction, image registration, and manual marking of stroke lesions by radiologists on the itk-snap platform. By multiplying the brain matrix and position matrix, the stroke regions for each area were obtained, and the proportion of stroke regions in the entire brain area was calculated. Subsequently, a machine learning model was established, utilizing a classification model based on the sparse representation method for feature selection and classification. The research results indicated that, on two independent test sets, the ACC of the cross-validation dataset using the Adboost method was 0.8519, while the ACC for the independent test set using the SRC method was 0.8125. This study suggests that the machine learning approach is effective in extracting and accurately quantifying features related to stroke prognosis, including vascular structure and stroke location (46).

The main information of the above included literatures is shown in Table 3.

**Table 3:** Summary of papers on machine learning for MRI on rehabilitation of Ischemic stroke.

PMID	15811787	26606516	33152045	33220140	34266905
YEAR	2005	2015	2020	2021	2022
LEARNING APPROACH	machine learning	machine learning	machine learning	machine learning	machine learning
PRIMARY AUTHOR	Christian Gottrup	Bum Joon Kim	Grosser M	Janne Hamann	Rob A van de Graaf
DISEASE	Acute Strok	Cerebral Infarction	acute ischemic stroke	stroke patients with middle cerebral artery-M1 occlusions and early thrombectomy	ischemic stroke
DATA VOLUME	magnetic resonance imaging of 14 patients with acute stroke	Thirty-five cases of ischemic infarction patients with visual field defects (VFD) due to posterior cerebral artery (PCA) infarction	weighted MRI data from 99 patients.	222 patients with acute ischemic stroke due to middle cerebral artery (MCA)-M1 occlusion who received EVT	A cohort of 3180 patients who successfully underwent reperfusion
DATA TYPE	mri	MRI	weighted MRI datasets	magnetic resonance imaging features.	data from the MR CLEAN Registry
METHODS	k-NN, Gaussian weighted, and constant radius search classification.	Support Vector Machine	global machine learning model	different machine-learning models and	A multivariate ordinal regression model
RESULTS	Optimal k-NN and Gaussian weighted algorithms exhibit no significant performance difference, yet both are notably superior to the constant radius implementation	The occurrence of left PCA infarction, pre-onset MRI duration, and reil in the tongue, corpus callosum, and cuneal cortex are indicative of a favorable prognosis for lateral visual field defects (VFD). When compared to clinical variables, the combination of rEIL in various cortical subregions demonstrates a superior predictive effect for lateral VFD. The inclusion of rEIL in other variables improves the prognosis prediction for lateral VFD.	The ensemble LR model performed optimally with the highest mean ROC AUC value (0.872±0.092), while the ensemble RF model excelled with the highest mean Dice coefficient (0.353±0.220).	The successful reperfusion rate reached 78%, with favorable outcomes observed in 54% of patients (Modified Rankin Scale score 0-2).	
CONCLUSIONS	The team has concluded that the IB method can be employed for predicting the ultimate infarction in patients with acute ischemic stroke. However, further efforts are necessary to make it applicable in a clinical setting.	The team has derived estimates of rEIL that provide valuable information about the location of ischemic lesions. rEIL accurately predicts significant improvements in VFD, and the conclusion is reinforced when combined with other variables, enhancing predictive capabilities.	Compared to a single global machine learning model trained on voxel information independent of brain location, a locally trained machine learning model provides more accurate predictions of lesion outcomes.	There exists a correlation between small infarct lesions and favorable functional outcomes, while age is associated with decreased chances of favorable outcomes and functional improvement. Standalone radiological information as a predictor for functional outcomes exhibits relatively poor performance. Upon incorporating imaging variables into patient characteristics, no significant differences were observed among the predictive variable sets.	
PMID	34218182	36504664	36143010	36055039	36804312
YEAR	2021	2022	2022	2022	2023
LEARNING APPROACH	machine learning	machine learning	machine learning	machine learning	machine learning
PRIMARY AUTHOR	Abedi V	Ahmed F Elsaid	Guo Y	Li Y	Luo Y
DISEASE	acute ischemic stroke	ischemic stroke	Ischemic Stroke	acute ischemic stroke	acute basilar artery occlusion
DATA VOLUME	Clinical data from a cohort of 7144 patients retrieved from the database.	360 ischemic stroke patients were enrolled, and a continued investigation was conducted with a subset of 354 individuals.	88 DSC-PWI images from 156 patients.	A total of 260 stroke patients undergoing mechanical thrombectomy at our hospital were randomly divided into a training set (n=182) and a test set (n=78) in a 7:3 ratio.	A total of 132 patients were randomly allocated into a training group (n = 106) and a test group (n = 26).
DATA TYPE	MRI	T2 diffusion-weighted MRI	DSC-PWI	DWI	dwi
METHODS	EHR	generalized additive modeling (GAM)	Ten machine learning models	Combining machine learning with radiomics features	radiomics-clinical machine learning model
RESULTS	The model's average AUROC ranges from 0.76 to 0.81, with the Random Forest model excelling in a one-month window (AUROC=0.82). Shorter prediction windows show high Negative Predictive Values (NPV), peaking at 91.1. The RF model has the highest Positive Predictive Value (PPV) in a six-month window (0.92), while the XGBoost-based model achieves the highest accuracy in a one-month window (precision of 0.89). Age, hemoglobin levels, and BMI consistently rank as the top three influential factors across different prediction windows, with average overall importance values of 96.3%, 68.2%, and 55.5%, respectively.	The rate of hemorrhagic transformation (HT) in ischemic stroke patients was 19.8%.	The R values of dimensionality reduction features (DRF) obtained through methods such as First_order, GLCM, GLDM, GLRRM, GLSZM, and NGTDM using PCA, ICA, t-SNE, ISOMAP, and UMAP were 0.110 ± 0.121, 0.140 ± 0.079, 0.110 ± 0.121, 0.294 ± 0.139, and 0.098 ± 0.133, respectively.	In the training set, the AUC for predicting post-mechanical thrombectomy outcomes was 0.945 (95% CI: 0.890–0.975), while in the test set, it was 0.920 (95% CI: 0.849–0.981).	The area under the ROC curve (AUC) of the radiomics-clinical model was 0.897 in the training group and 0.935 in the test group. For the radiomics model alone, the AUC was 0.887 in the training group and 0.840 in the test group. The clinical model achieved an AUC of 0.746 in the training group and 0.766 in the test group.
CONCLUSIONS	The machine learning model successfully predicted outcomes for stroke patients across different time periods and highlighted the crucial role of these factors in predicting mortality rates.	The team identified cerebral microbleeds, NIHSS, and infarct size as predictors of HT. The optimal predictive models were RFC and GBC, revealing the ability to capture non-linear interactions among predictive factors.	In the detection, assessment, and outcome prediction of ischemic stroke, employing various feature extraction and dimensionality reduction methods can achieve satisfactory performance.	A model based on radiomic and machine learning features exhibits high predictive efficiency for the prognosis of acute ischemic stroke after mechanical thrombectomy.	The radiomics-clinical machine learning model based on DWI demonstrated satisfactory performance in predicting preoperative ineffective reperfusion in ABAO patients.
PMID	37416313	36650639	36699499	37745661	36934582
YEAR	2023	2023	2023	2023	2023
LEARNING APPROACH	machine learning	machine learning	machine learning	machine learning	machine learning
PRIMARY AUTHOR	Xu Y	Wang X	Yu H	Lee J	Weng S
DISEASE	corpus callosum infarction	acute ischemic stroke	acute ischemic stroke	acute ischemic stroke	ischemic stroke
DATA VOLUME	A total of 314 patients with acute corpus callosum infarction (CC)	A total of 573 patients were included in the study, comprising 398 with small-vessel occlusion (SVO) and 175 with large artery atherosclerosis (LAA) acute ischemic stroke (AIS).	148 patients with acute ischemic stroke due to anterior circulation artery occlusion.	3,687 patients were in one group, and there were 250 and 110 patients in the other two groups for validation.	CTA and MRI images from 97 patients
DATA TYPE	Neuroimaging modalities such as MRI, MRA, or CTA were employed for neuroimaging assessments	DWI	multi-modal MRI radiomics	MRI and associated clinical information.	CTA、DWI
METHODS	interpretable machine learning-derived early warning strategy	Logistic regression (LR) and machine learning (ML)	machine learning based on multi-modal MRI radiomics	ML algorithms	machine learning-based method
RESULTS	The predictive performance of the LR model for SCD after CC infarction surpasses that of the other six machine learning models. Through LASSO and SHAP analyses, nine key predictive factors were identified in the LR model output. Additionally, factors independently associated with cognitive outcomes were revealed.	In brief, the LR model for small-vessel occlusion acute ischemic stroke (SVO-AIS) demonstrated excellent outcome AUC of 0.86 [0.78–0.94] and good outcome AUC of 0.88 [0.8–0.96]. For the LR model in large artery atherosclerosis acute ischemic stroke (LAA-AIS), the AUCs were 0.73 [0.54–0.91] for excellent outcomes and 0.75 [0.59–0.91] for good outcomes. The GPR model for SVO-AIS exhibited AUCs of 0.86 [0.77–0.95] for excellent outcomes and 0.86 [0.77–0.96] for good outcomes, while the GPR model for LAA-AIS had AUCs of 0.65 [0.47–0.83] for excellent outcomes and 0.66 [0.49–0.84] for good outcomes. The GOA-RF model for SVO-AIS achieved AUCs of 0.85 [0.75–0.94] for excellent outcomes and 0.84 [0.74–0.94] for good outcomes. The GOA-RF model for LAA-AIS showed AUCs of 0.66 [0.49–0.84] for excellent outcomes and 0.68 [0.51–0.86] for good outcomes. The GOA-XGBoost model displayed AUCs of 0.87 [0.79–0.96] for excellent outcomes and 0.85 [0.76–0.94] for good outcomes, with AUCs of 0.91 [0.84–0.97] for excellent outcomes and 0.90 [0.83–0.97] for good outcomes in the LAA-AIS population.	Among 148 patients, 83 (56.1%) had a favorable prognosis, while 65 (43.9%) had an unfavorable prognosis. The accuracy of the SVM model was 79%, the RF model was 82%, the LightGBM model was 83%, the CatBoost model was 81%, and the XGBoost model was 80%.	In the patient cohort, 30.4% experienced unfavorable outcomes, with external validation data showing rates of 37.2% and 29.1%, respectively. The internal test set, external validation sets A and B demonstrated AUROC values of 0.790, 0.791, and 0.873, while the Brier scores were 0.172, 0.202, and 0.141, respectively.	On two independent test sets, the accuracy (ACC) for the cross-validated dataset using the Adboost method was 0.8519, while the ACC for the independent test set using the SRC method was 0.8125.
CONCLUSIONS	The combination of the LR model and SHAP interpreter can assist in achieving personalized risk predictions and, given its suboptimal long-term efficacy, may serve as a decision tool for early intervention.	Various small vessel disease (SVD) markers carry different prognostic weights in acute ischemic stroke (AIS) patients. Only the SVD burden accurately predicts the prognosis of small-vessel occlusion acute ischemic stroke (SVO-AIS) patients.	The potential value of a multimodal radiomic approach in accurately predicting clinical outcomes in patients with acute ischemic stroke (AIS). It aids in preventing mental disorders following a stroke.	With the aid of the SHAP method, we can attain an in-depth understanding of how critical features contribute to model predictions and how changes in these features influence such predictions	This machine learning approach can effectively explore and accurately quantify features related to stroke prognosis, including vascular structure and stroke location.

#### 2.5.2 The application of deep learning in stroke

In 2018, Nielsen et al. conducted a detailed analysis of a multi-center study involving 222 patients from the I-KNOW consortium and remote ischemic pre-conditioning for MRI. The study included a subgroup of patients receiving intravenous rtPA treatment (*n* = 187) and the imaging results of 35 untreated patients. In the training phase, a CNN was trained using TensorFlow 2.7.9 to automatically identify initial arterial input functions. Deconvolution was performed using concentration curve parameters to obtain average microvascular transit time and cerebral blood flow. CNNTmax was used to assess the accuracy of Tmax and was compared with regression methods using generalized linear models (GLM) to predict the risk of infarction at the individual voxel level. The final infarction prediction performance was evaluated by AUC from T2-FLAIR scans. Patients receiving intravenous treatment were divided into independent training (158 cases) and test sets (29 cases) to evaluate the model’s performance in independent patients. Follow-up infarctions were assessed by four radiology experts 1 month after stroke. The 35 untreated patients underwent post-training, resulting in CNNdeep-rtPA. Differences were assessed through post-training of CNNdeep. The new model was evaluated on 29 patients in the trial group who received intravenous rtPA treatment, and AUC and final infarct area were compared. The results showed that CNNshallow tended to overestimate the final lesion volume, while CNNTmax predicted a lower risk of infarction. In contrast, CNNdeep provided better visual predictions. CNNdeep had an AUC of 0.88 ± 0.12, significantly better than CNNshallow (0.85 ± 0.11), GLM (0.78 ± 0.12), CNNTmax (0.72 ± 0.14), and ADCthres (0.66 ± 0.13). There was a significant difference between CNNdeep and GLM (*p* = 0.005), CNNTmax (*p* < 0.003), and ADCthres (*p* < 0.0001), while the difference between CNNdeep and CNNshallow was not significant (*p* = 0.063) (79).

In 2018, Pinto et al. included MRI images and clinical information from 75 ischemic stroke patients who underwent mechanical thrombectomy from the ISLES2017 dataset. Using a Deep Learning architecture that combines U-net with two-dimensional Gated Recurrent Units (GRU), the study integrated clinical information at the population level and analyzed it using the Thrombolysis in Cerebral Infarction (TICI) scale. The study experimented with cross-validation on the training set and compared its results with a baseline architecture that did not include any clinical metadata. This research innovatively combined imaging and non-imaging clinical data in a Deep Learning architecture and, through the development of a customized loss function, incorporated clinical information in both the learning and prediction phases. This approach more accurately predicted different outcome scenarios. The study evaluated the performance of the method using five metrics (Dice similarity coefficient, accuracy, recall, Hausdorff distance, and average symmetric surface distance). In the end, the method achieved a Dice score of 0.29 ± 0.22, Hausdorff distance of 47.17 ± 22.13, ASSD of 7.20 ± 4.14, precision of 0.26 ± 0.23, and recall of 0.61 ± 0.28 (80).

In 2019, King et al. included MR images from 444 patients. They employed a multi-atlas skull stripping algorithm for skull stripping. By aligning brain images to the centerline of the axial plane, the study proposed a Deep Learning model, compared it with several benchmark models, and introduced an improved model. The performance of these models was evaluated through multiple validations, using metrics such as AUC, accuracy, and overlap coefficient. Here are the AUC and overlap coefficient results: 2D CNN: AUC of 0.783 ± 0.030, overlap coefficient of 0.728. 3D CNN: AUC of 0.799 ± 0.029, overlap coefficient of 0.717. Unitary CNN-Opposite: AUC of 0.871 ± 0.024, overlap coefficient of 0.811.SR-KDA: AUC of 0.788 ± 0.031, overlap coefficient of 0.679. Results for accuracy and recall are as follows: 2D CNN: Accuracy of 0.211, recall of 0.700. 3D CNN: Accuracy of 0.220, recall of 0.693. Unitary CNN-Opposite: Accuracy of 0.222, recall of 0.799. SR-KDA: Accuracy of 0.671, recall of 0.171. Experimental evidence suggests its optimal performance when trained with opposite patches. Through visualizing the results of the deep CNN, the study provides a detailed analysis of the model’s performance in large infarcts and specific scenarios. Overall, this deep CNN is considered the best tissue outcome prediction model with significant performance advantages (81).

In 2020, Yu et al. included 182 patients with acute ischemic stroke for MRI analysis. Initially, segmentation of T2-weighted fluid-attenuated inversion recovery images was performed by neuroradiologists. Subsequently, image registration and normalization were carried out using SPM12 software, and preprocessing of images was done with the Tmax and ADC segmentation from RAPID software. The study employed a neural network with U-Net architecture and calculated various performance metrics, including DSC, Positive Predictive Value (PPV), sensitivity, specificity, lesion volume error, etc., for the Deep Learning model, Tmax, and ADC threshold methods. Data analysis was conducted using Stata version 0.70. In this study, the Deep Learning model exhibited a median area under the curve of 0.92 (IQR, 0.87–0.96). Using a threshold of 0.50, the median DSC overlap for this model was 0.53 (IQR, 0.31–0.68), with a sensitivity of 0.66 (IQR, 0.38–0.86), specificity of 0.97 (IQR, 0.94–0.99), PPV of 0.53 (IQR, 0.28–0.74), volume error of 9 mL (IQR, −14-29), and absolute volume error of 24 mL (IQR, 11–50). The study concluded that the Deep Learning model appears to successfully predict infarct lesions from baseline imaging without the need for reperfusion information and performs comparably to existing clinical methods (82).

In 2020, Debs et al. included 109 patients with cerebral arterial occlusion who underwent thrombectomy for MRI and follow-up FLAIR imaging. They employed Olea Sphere software for circular singular value decomposition, extracting parameter maps from DSC-PWI images. In image processing, FSL was used to remove the skull and normalize the images, ensuring standardization across patients. Experts manually labeled lesions on baseline DWI and final FLAIR images using 3D Slicer. Three models were established: a “universal” model trained on the entire cohort without considering reperfusion status, a “reperfusion” model trained only on reperfusion patients, and a “non-reperfusion” model. These models, based on the U-Net architecture, received five inputs (DWI, ADC, Tmax, CBF, and CBV) and generated probability maps for lesions, healthy tissue, and background. The final infarct was defined by setting a threshold of 0.5. Result assessments included metrics such as DSC, accuracy, recall, volume similarity (*VS*), HD, and AUC. In non-reperfusion patients, the non-reperfusion model predicted an infarct volume of 39.7 mL with a DSC of 68%. In reperfusion patients, the reperfusion model predicted an infarct volume of 17.5 mL with a DSC of 89%. CNN-based models demonstrated excellent AUC values, with 0.87 for reperfusion patients and 0.81 for non-reperfusion patients. The study concluded that incorporating reperfusion status into training enhances model performance, and CNN outperforms clinical models. Predicting the final infarct plays a crucial role in evaluating treatment efficacy (83).

In 2020, Osama et al. included DWI and PWI data from 43 patients with acute ischemic stroke. They constructed a neural network model based on multi-parameter feature embedding (PMFE-SN) and applied it to predict the outcomes of acute ischemic stroke treatment. By preprocessing the images and employing two twin convolutional neural networks to build Siamese networks, effective feature extraction for samples of the same or different categories was achieved. At the output layer, the extracted features were normalized using cosine similarity, and training was performed with the backpropagation algorithm and stochastic gradient descent to minimize the binary cross-entropy loss function. The study extensively used various evaluation metrics, including Mean Absolute Error (MAE), macro-average F1 (F1macro), macro-average precision (Pmacro), macro-average recall (Rmacro), Matthews Correlation Coefficient (MCC), and AUC. The results showed a significant improvement in evaluation metrics for PMFE-SN compared to traditional random forest methods. Pmacro increased from 0.152 to 0.258, Rmacro increased from 0.21 to 0.31, F1macro increased from 0.18 to 0.28, and MCC increased from 0.04 to 0.09. The overall AUC value increased from 0.50 for the random forest method to 0.81. The research conclusion explicitly stated that PMFE-SN demonstrated excellent performance in predicting categories with both few and numerous samples (84).

In 2021, Bo et al. randomly selected 50 patients admitted to the hospital from January 2019 to January 2021. They conducted an analysis of the impact features of MRI on critically ill patients with cerebral infarction using CNN and explored the clinical application of Artificial Intelligence-assisted systems in imaging. Additionally, they established a CNN Artificial Intelligence system for learning and training, utilizing the CNN system to extract data such as pixel grayscale statistics, regional feature descriptions, and local region gradient analysis. The data were then computed using computer technology. Comparing the segmentation results, it was found that the segmentation Dice coefficient of U-Net without additional supervision was 81.74 ± 0.40%, and P-Net’s Dice coefficient was 86.39 ± 0.31%. In the first stage, DPA-UNet was 83.52 ± 0.31%, in the second stage, it was 88.29 ± 0.27%, and in the third stage, it was 91.74 ± 0.12%. There was no significant difference between the data sets. A higher Dice coefficient indicates more accurate segmentation. Through the analysis of T1WI, contrast-enhanced T1WI, and T2WI images, significant differences were found between GLSZM and ALL, GLRLM, MGLSZM, and GLSZM (85).

In 2021, Ma et al. selected 36 patients diagnosed with lacunar cerebral infarction (LCI) between February 2019 and June 2020 as the study subjects. The objective was to explore the MRI features using the fuzzy local information C-means clustering (FLICM) image segmentation method and to analyze the risk factors for recurrent stroke in patients with lacunar infarction. The study, based on the FLICM algorithm, introduced the Canny edge detection algorithm, and Fourier shape descriptors to optimize the algorithm. The research investigated the differences in Jaccard coefficient, Dice coefficient, peak signal-to-noise ratio (PSNR), structural similarity index measure (SSIM), processing time, and segmentation accuracy between the optimized FLICM algorithm and other algorithms when segmenting brain tissue MRI images. Patients were categorized into a control group (no recurrent stroke, 20 cases) and a stroke group (recurrent stroke, 16 cases) based on whether they experienced another stroke. The study compared the differences in MRI features between the two groups and utilized logistic multivariate regression analysis to identify risk factors for recurrent stroke after lacunar infarction. The results showed that, under the same noise conditions, the optimized FLICM algorithm exhibited higher Jaccard coefficient, Dice coefficient, PSNR, and SSIM values when segmenting brain tissue compared to other algorithms. Additionally, age and a history of hypertension were identified as risk factors for recurrent stroke after lacunar infarction (86).

In 2021, Tolhuisen et al. included 316 FU-DWI (Follow-Up Diffusion Weighted Imaging) data from the HERMES, ISLES, and MR CLEAN-NOIV databases. They transformed DWI images into standard MNI space using the SPM8 toolbox. A Deepmedic network was trained on the DWI images from the HERMES dataset, with the data split into training set (70%), validation set (10%), and test set (20%). The trained network was then applied to other datasets. Simultaneously, they manually adjusted using ITK-SNAP, and developed and optimized a Convolutional Autoencoder (CAE) using the Keras library. The study aimed to accurately predict functional independence within 90 days using mRS scores. They optimized SVM by adjusting the kernel type, coefficient (gamma), and regularization parameter (C). Feature normalization was performed on all features using scikit-learn’s “RobustScaler” function, with 80% of DWI images used for 5-fold cross-validation. The remaining 20% of images were used to test the final classifier’s performance by evaluating AUC of ROC curve. DeLong’s test was applied for pairwise comparisons and to test for statistical differences. The study results demonstrated that the AUC values for the CAE and radiomics feature-based classifier were 0.88 and 0.81, respectively, while the FIV-based classifier had an AUC value of 0.79. The SVM classifier based on radiomics features achieved the highest accuracy of 0.80, whereas the SVM classifier based on FIV had the highest recall of 0.73 (87).

In 2022, Zeng et al. included 711 ischemic stroke patients admitted between March 1, 2017, and December 31, 2020, as the training group. Additionally, they included 140 ischemic stroke patients admitted to the same hospital between January 1, 2021, and May 1, 2021, as the testing group. Patients were assessed with NIHSS scores on admission and on the seventh day (classified as stage 1 if NIHSS <5 and stage 2 if NIHSS ≥5). The testing group underwent MRI within 24 h to 7 days after a subacute stroke episode and received thrombolysis. The researchers converted DICOM DWI to NIfTI format, removed DWI artifacts, corrected image alignment using 3Dslicer, normalized images, and adjusted pixel size using SimpleITK. The data were divided into eight models (Models A-H) based on admission NIHSS and NIHSS on the seventh day. They employed 3D CNN models (Models E-G) based on input DWI with pixel and preprocessing strategies. The performance of different models was compared using the DeLong test (*p* < 0.05, statistically significant), and AUC was evaluated after exporting results. Model E demonstrated the highest AUC in the testing set, particularly in predicting NIHSS stage on admission. Model A performed best in predicting NIHSS stage on admission when a subsequent ischemic stroke occurred, with Model D correctly predicting all cases of subsequent ischemic stroke in later cycles. For predicting NIHSS stage on the seventh day of hospitalization, Model E had the highest AUC, with relatively higher AUC in predicting NIHSS stage on the seventh day in patients with anterior circulation ischemic stroke and relatively lower AUC in predicting NIHSS stage on the seventh day in patients with posterior circulation ischemic stroke (88).

In 2022, Wong et al. included 875 patients with acute ischemic stroke (700 in the training set and 175 in the testing set) with DWI and MRI data. They manually segmented acute infarct volumes using MRIcro, selecting eADC to confirm the diffusion-restricted portion of the infarct, and extracting image volumes in Matlab using SPM12 tools. Subsequently, they employed a Deep Learning model, training a group convolutional neural network with U-Net architecture and Dice loss function, incorporating augmentations like rotation and reflection for segmentation. Evaluation metrics such as Dice scores, precision, and recall were compared with manual labels. Additionally, a multivariate logistic regression model was constructed to assess the predictive impact of topological infarct volume on 90-day mRS outcomes. In the testing data, the model without data augmentation achieved a Dice score of 0.85, precision of 0.83, and recall of 0.89. The model with data augmentation had a Dice score of 0.84, precision of 0.84, and recall of 0.89. Using each output for 30 fine brain regions to predict the mRS achieved an AUC of 0.80 and accuracy of 0.75 (89).

In 2022, Nazari-Farsani et al. included 455 patients with acute ischemic stroke, and obtained DWI and PWI images. They initially preprocessed the images using SPM12 software and then applied a 3D Attention Gated (AG) U-net model with Rectified Linear Unit (ReLU) activation function and ADAM optimizer. The model was subjected to 5-fold cross-validation using a hybrid loss function. The evaluation metrics included AUC, sensitivity, specificity, DSC, volume error, absolute volume error, and Jaccard index. Statistical analysis was conducted using the Scipy package in Python. The results showed that the median AUC of the DCNN model was 0.91 (IQR: 0.84–0.96). Using a probability threshold of 0.5, the median sensitivity, specificity, and Jaccard index were 0.60 (IQR: 0.16–0.84), 0.97 (IQR: 0.93–0.99), and 0.50 (IQR: 0.21–0.70), respectively. The DCNN model’s median DSC was 0.50, while the ADC threshold method had a median DSC of 0.18 (*p* < 0.01). The predicted volume by the model exhibited a high correlation with the actual lesion volume, with a correlation coefficient of 0.73 (*p* < 0.001) (90).

In 2022, Moulton et al. included patients suspected of large vessel occlusion and candidates for reperfusion therapy with DWI. They preprocessed the images using techniques such as skull stripping, manual correction, and normalization. High-level feature sets were generated using VGGNet, and the model was trained and evaluated through internal training-validation sets and LOCO cross-validation. Adam optimizer and binary cross-entropy loss function were employed during model training. The analysis involved 322 patients, with 113 from the Pitié-Salpêtrière registry, 94 from the Insulin Stroke Trial, and 115 from six centers in the ASTER trial. Significant differences were found in stroke-to-needle time (*p* = 0.008) and stroke side (*p* < 0.001). The Deep Learning ensemble model performed the best, with an accuracy of 0.79, AUC of 0.83, sensitivity of 0.67, specificity of 0.87, PPV of 0.79, and NPV of 0.78. The conclusion suggests that the model has potential applications in predicting long-term functional outcomes in stroke patients and could be used as a patient stratification strategy for neuroprotective and rehabilitation therapies (91).

In 2023, Lv et al. included 282 patients with 50% stenosis of the internal carotid artery for MRI 1 week later. They preprocessed DICOM images, applied convolution, utilized max-pooling layers, and fully connected layers to output images. Features from different modalities were concatenated, and a Fully Connected (FC) layer was created in the corresponding dimension of the channel to obtain classification results. In statistics, the loss function allows the evaluation of the difference between the true and predicted values. They measured the performance of common machine learning methods based on the random forest, logistic regression, and XGBoost concepts in predicting recurrent stroke. The models were trained to minimize the differences between model values and actual values. Results showed that the AUC values for four different modalities were 62.2, 68.9, 65.4, and 60.4%, respectively. The AUC for T2WI modality was 8.5% higher than that for the ADC modality. ADC modality performed relatively worse, being 11.6% lower than the FLAIR modality, which exhibited better performance. The AUC values for the three algorithms were 50.6, 64.8, and 66.8%, with XGBoost achieving an AUC of 66.8% (92).

In 2023, Ye et al. conducted a study involving 441 patients with acute ischemic stroke. They utilized MRI and grouped the patients based on the prognosis NIHSS scores. The ITK-SNAP 6.0.3 software was employed to independently segment ROIs in the images, generating three-dimensional structural data of the lesions. Subsequently, radiomic features of each annotated lesion were extracted using radiomics analysis tools (Pyradiomics software package), resulting in a total of 17 clinical features and 851 radiomic features. After preprocessing steps such as data imputation, denoising, standardization, filtering, concatenation, and balancing, they constructed a multi-level cascaded Deep Learning ensemble (EDL) model, combining ensemble learning and Deep Learning. The optimized Deep Learning ensemble (OEDL) model was established by introducing the big bang optimization algorithm (BBOA). Model training was carried out on a Linux workstation equipped with a GPU, utilizing the Python 3.7 platform and TensorFlow 2.8 framework, with 70% of the data allocated for the training set and 30% for the test set. The statistical analysis of clinical, radiomic, performance prediction, and comparative results showed that Macro-AUC, ACC, Macro-R, Macro-P, and Macro-F1 achieved values of 97.89, 95.74, 94.75, 94.03, and 94.35%, respectively. In comparison, the EDL method demonstrated a Macro-AUC of 96.68% and ACC of 92.55%. The OEDL method achieved a Macro-AUC of 97.89% and ACC of 95.74%. The SMOTEENN-based mixed sampling method exhibited the best classification performance, with Macro-AUC, ACC, Macro-R, Macro-P, and Macro-F1 reaching 97.89, 95.74, 94.75, 94.03, and 94.35%, respectively (93).

The main information of the above included literatures is shown in Table 4.

**Table 4:** Summary of papers on deep learning for MRI on rehabilitation of ischemic stroke.

PMID	29720437	30568631	31131293	32163165
Year	2018	2018	2019	2020
Learning approach	Deep learning	Deep learning	Deep learning	Deep learning
Primary author	Nielsen A	Pinto A	Ho KC	Yu Y
Disease	Acute ischemic stroke	Stroke lesion	Ischemic stroke	Acute ischemic stroke
Data volume	A total of 222 patients were included, with 187 receiving rtPA treatment (recombinant tissue-type plasminogen activator).	75 ischemic stroke patients divided into two groups: training (*n* = 43) and testing (*n* = 32),	444 patient MR images were retrieved and examined from the University of California-Los Angeles picture archiving and communication system between December 2005 and December 2015.	182 patients with acute ischemic stroke, in accordance with the conventions of scientific literature.
Data type	MRI	MRI	MRI, Handcrafted features derived from perfusion images.	MRI
Methods	CNNdeep	Adopting a deep learning architecture that combines U-net with two-dimensional Gated Recurrent Units (GRU), following the pattern of nature.	Deep convolution neural networks (CNNs)	Neural network
Results	The AUC for CNNdeep is 0.88, surpassing the generalized linear model with an AUC of 0.78, CNNTmax with an AUC of 0.72, ADCthres with an AUC of 0.66, and substantially outperforming CNNshallow with an AUC of 0.85.	In accordance with the conventions of Nature: Dice Similarity Coefficient (DSC): Baseline 0.34 ± 0.22, Proposal 0.35 ± 0.22; Hausdorff Distance: Baseline 35.09 ± 17.27, Proposal 31.38 ± 15.81; Average Symmetric Surface Distance (ASSD) series: Baseline 6.08 ± 5.27, Proposal 5.55 ± 5.00; Precision: Baseline 0.37 ± 0.29, Proposal 0.41 ± 0.30; Recall: Baseline 0.54 ± 0.26, Proposal 0.47 ± 0.24.	Our deep CNN model improves feature learning, achieving an AUC of 0.871 ± 0.024, outperforming existing models for tissue fate.	The curve of 0.92, DSC of 0.53, and volume error of 9 mL.
Conclusions	The notable improvement in prediction accuracy enhances the potential for automated decision support, offering personalized treatment plan recommendations, surpassing the current state-of-the-art.	Leveraging deep learning for stroke outcome prediction, the study demonstrates promising outcomes on the ISLES 2017 dataset while indicating avenues for potential enhancements in clinical applications.	Our study utilizes deep learning techniques for predicting stroke tissue outcomes, advancing magnetic resonance imaging perfusion analysis toward becoming an operational decision support tool for guiding stroke treatment.	The deep learning model accurately predicted infarct lesions without reperfusion information, performing similarly to current clinical methods.
PMID	33450521	33105609	34385896	34887708
Year	2021	2020	2021	2021
Learning approach	Deep learning	Deep learning	Deep learning	Deep learning
Primary author	Debs N	Osama S	Bo Y	Chunli Ma
Disease	Acute ischemic stroke	Acute ischemic stroke	Cerebral infarction	Lacunar cerebral infarction
Data volume	109 patients, including 35 without reperfusion.	43 samples from the ISLES 2017 dataset.	50 patients with cerebral infarction were selected randomly.	36 patients with lacunar myosphere infarction (no recurrence in the control group, *n* = 20, recurrence in the stroke group, *n* = 16).
Data type	Baseline diffusion and perfusion-weighted magnetic resonance imaging (MRI)	DWI、PWI	MRI	MRI
Methods	Convolutional neural networks (CNN)	Parallel multi-parametric feature embedded siamese network (PMFE-SN)	Convolutional neural network (CNN)	Deep learning algorithm
Results	The peak values of DSC (Dynamic Susceptibility Contrast) in reperfused and non-reperfused patients were 0.44 ± 0.25 and 0.47 ± 0.17, respectively. The Area Under the Curve (AUC) for reperfused patients was 0.87 ± 0.13, while for non-reperfused patients, it was 0.81 ± 0.13. The AUC for the perfusion-diffusion mismatch model was 0.73 ± 0.14.	PMFE-SN exhibits a significant improvement compared to traditional random forest methods. Pmacro increased from 0.152 to 0.258, Rmacro improved from 0.21 to 0.31, F1macro rose from 0.18 to 0.28, and MCC increased from 0.04 to 0.09. The overall AUC value elevated from 0.50 with the random forest method to 0.81.	In the absence of additional supervision, U-Net exhibited a segmentation Dice coefficient of 81.74 ± 0.40%, while P-Net demonstrated a Dice coefficient of 86.39 ± 0.31%. The first stage of DPA-UNet yielded a Dice coefficient of 83.52 ± 0.31%, the second stage achieved 88.29 ± 0.27%, and the third stage reached 91.74 ± 0.12%. There were no significant differences observed among the data sets.	The optimized FLICM algorithm, under the same noise conditions, exhibits higher Jaccard coefficient, Dice coefficient, PSNR, and SSIM values in the segmentation of brain tissues compared to other algorithms. Furthermore, age and a history of hypertension are identified as risk factors for recurrent strokes following lacunar infarction.
Conclusions	Utilizing a convolutional neural network (CNN)-based model demonstrates superior performance compared to the perfusion-diffusion mismatch model commonly employed in clinical settings.	PMFE-SN demonstrates exceptional performance in predicting categories with both fewer and more samples, delving into pre-and post-treatment clinical data. Exploring the use of additional similarity metrics in this context could contribute to a comprehensive enhancement of predictive accuracy for outcomes in acute ischemic stroke treatment.	Utilizing CNN to analyze features in MRI images of critically ill cerebral infarction patients, we have identified an image diagnostic method that mitigates subjective visual judgment errors to a certain extent. The introduction of a deep supervision mechanism enhances the recognition capabilities of U-Net, holding significant importance for the accurate extraction and reconstruction of MRI images in patients with cerebral infarction.	The optimized FLICM algorithm demonstrates effective segmentation of brain MRI images, with age and a history of hypertension identified as risk factors for recurrent strokes in lacunar infarction patients. This study provides valuable insights for the diagnosis and prognosis of lacunar infarction
PMID	35892499	35887776	35545938	36481696
Year	2022	2022	2022	2023
Learning approach	Deep learning	Deep learning	Deep learning	Deep learning
Primary author	Tolhuisen ML	Zeng Y	Wong KK	Nazari-Farsani S
Disease	Acute ischemic stroke	Ischemic stroke	Acute ischemic stroke	Ischemic stroke
Data volume	316 follow-up DWI datasets sourced from the HERMES, ISLES, and MR CLEAN-NOIV databases.	851 patients (711 in the training set and 140 in the test set)	875 patients (*n* = 700 in the training group, *n* = 175 in the test group)	445 patients
Data type	FU-DWI	DWI	MRI, DWI	PWI
Methods	Deep learning network	CNN	A rotation-reflection equivariant model was developed based on U-Net and grouped convolutions.	Deep convolutional neural network (DCNN)
Results	The AUC values for the CAE and radiomic features classifier are 0.88 and 0.81, respectively, while the classifier based on FIV achieves an AUC value of 0.79. The SVM classifier based on radiomic features attains the highest accuracy at 0.80, whereas the SVM classifier based on FIV achieves the highest recall at 0.73.	Following the conventions of scientific writing: The proposed model exhibits improved performance in predicting NIHSS stages on the 7th day of hospitalization compared to admission (best AUC 0.895 vs. 0.846). Model D, trained on DWI images, achieved the best AUC of 0.846 in predicting NIHSS stages at admission. Model E, also trained on DWI images, achieved the best AUC of 0.895 in predicting NIHSS stages on the 32nd day of hospitalization. The model demonstrates favorable performance in predicting NIHSS stages on the 7th day of hospitalization for both anterior and posterior circulation strokes, with best AUCs of 7.0 and 905.0, respectively.	The segmentation model achieved Dice scores of 0.88 (training) and 0.85 (testing). The AUC for predicting modified Rankin Scale outcomes based on refined stroke volumes in 30 brain regions was 0.80, with an accuracy of 0.75.	The model achieved a median AUC of 0.91. Using a threshold of 0.5 for infarction probability, median sensitivity and specificity were 0.60 and 0.97 respectively, while the median DSC was 0.50, and the absolute volume error was 27 mL.
Conclusions	The prediction of functional outcomes should not solely rely on FIV; FU-DWI images should capture additional prognostic information	Our 3D-CNN model efficiently predicts stroke-related neurologic damage using DWI images, demonstrating outstanding performance in predicting NIHSS stages on the 7th day of hospitalization. It holds potential clinical decision-making value in subgroup analysis.	We developed a rotation-reflection equivariant deep learning model to effectively segment acute ischemic stroke lesions in diffusion-weighted imaging, showcasing competitive performance in well-balanced testing cases across different vascular territories. Moreover, when integrated with clinical factors, the model exhibited high accuracy and AUC in predicting 90-day modified Rankin Scale outcomes.	An AG-DCNN using diffusion information alone upon admission was able to predict infarct volumes at 3–7 days after stroke onset with comparable accuracy to models that consider both DWI and PWI.
PMID	36169033	36908778	37416306	
Year	2023	2023	2023	
Learning approach	Deep learning	Deep learning	Deep learning	
Primary author	Moulton E	Lv P	Ye W	
Disease	Post-stroke	Carotid atherosclerotic stenosis	Acute ischemic stroke	
Data volume	322 patients from the ASTER and INSULINFARCT trials as well as the Pitié-Salpêtrière registry.	Patients with 50% stenosis in 282 internal carotid arteries	441 patients with acute ischemic stroke, 17 clinical features and 19 radiomic features were included.	
Data type	DWI	MRI	Clinical and radiomics features	
Methods	Convolutional neural networks (CNN)	Multi-modality fused network	Optimized ensemble of deep learning (OEDL) method.	
Results	The deep learning model demonstrated a notably superior performance with an area under the curve (AUC) of 0.83, surpassing lesion volume (AUC = 0.78) and ASPECT (AUC = 0.77). Upon aligning all classifiers with the specificity of the deep learning model (0.87), CNN’s sensitivity (0.67) significantly outperformed lesion volume (0.48) and ASPECT (0.50).	The AUC values for four different modalities were 62.2, 68.9, 65.4, and 60.4% respectively, with the AUC for T2WI modality surpassing that of the ADC modality by 8.5%. The ADC mode exhibited relatively poorer performance, being 11.6% lower than the more favorable FLAIR mode. The AUC values for three algorithms were 50.6, 64.8, and 66.8%, with the XGBoost classification algorithm achieving an AUC of 66.8%	The OEDL method with combined features and mixed sampling achieved the best classification performance, with 97.89, 95.74, 94.75, 94.03, and 94.35% for Macro-AUC, ACC, Macro-R, Macro-P, and Macro-F1	
Conclusions	The attention mechanism revealed that the network learned to naturally attend to the lesion to predict outcome.	Deep learning models demonstrate enhanced predictive capability for clinical symptomatology and outcomes in stroke patients.	The proposed OEDL method significantly enhances stroke prognosis prediction, outperforming models based on individual clinical or radiomic features. The combined data modeling approach demonstrates superior effectiveness and holds promise for guiding interventions.	

### 2.6 The potential applications of other imaging modalities in ischemic stroke

#### 2.6.1 Positron emission tomography

Positron emission tomography scans, employing oxygen-15 technology, provide information about glucose and oxygen metabolism abnormalities, ranging from the penumbra to ischemic tissue (94). With PET, it is possible to assess CBF reserve capacity in carotid atherosclerosis, aiding in the planning of future intervention strategies (95). In addition, TSPO PET can provide detailed information about metabolic and molecular changes during the neuroinflammatory phase after a stroke. However, the gold standard for TSPO PET quantification involves a 90-min scan and continuous arterial blood sampling, which is undoubtedly challenging in routine clinical practice. Artem Zatcepin and colleagues developed a machine learning-based algorithm to establish a simplified TSPO quantification method that can be easily implemented in clinical settings (96). Measurements of brain metabolism can provide new kinds of data for deep learning models, especially those based on computer vision. By combining metabolic and anatomic information, we can provide a more comprehensive picture of brain injury and build a more accurate predictive model of recovery. The detection of the brain metabolism of patients during rehabilitation can not only provide potential information support for the adjustment of rehabilitation measures, but also provide data sources for the construction of deep learning models containing temporal information.

#### 2.6.2 Single photon emission computed tomography

Ongoing advancements in SPECT instrumentation have facilitated the clinical application of several new technologies, including semiconductor Cadmium Zinc Telluride (CZT) detectors, absolute quantification of radiopharmaceutical uptake, multi-bed position whole-body SPECT acquisition, and novel non-parallel-hole collimators (97). The application of SPECT allows for the detection of cerebral blood flow reserve capacity in patients with carotid atherosclerotic disease, aiding in the formulation of future intervention plans (95). Using the acetazolamide challenge test, SPECT can also assess the decline in vascular reserve function. This information can be used to predict whether patients undergoing carotid endarterectomy will experience ischemia. SPECT is capable of characterizing the content of atherosclerotic plaques, including oxidized low-density lipoprotein and apoptotic bodies (98). Furthermore, SPECT can provide detailed information about metabolic and molecular changes (99). Based on these characteristics, SPECT has long been used for the evaluation of brain ischemia recovery (100). SPECT is currently used to explore the mechanism by which thalamic injury leads to a decline in word retrieval ability during brain ischemia recovery (101). Similarly, SPECT is also used to assess mid-term motor recovery after cerebral infarction (102). In summary, AI models based on SPECT have the potential for quantitative assessment of recovery progress.

#### 2.6.3 Dual-energy computed tomography

There has been some progress in the application of DECT in ischemic stroke. In a study by Na-Young Shin and colleagues, it was demonstrated that the collateral circulation status recorded by DECT could serve as a useful indicator for predicting the clinical prognosis of acute stroke patients (103). Additionally, Wang et al. (104) confirmed that DECT has certain value in the early diagnosis and prediction of intracranial hemorrhage after mechanical thrombectomy in patients with acute ischemic stroke. Furthermore, due to the advantages of dual-energy CT in measuring bone and muscle, it can be used to assess the recovery of motor function in stroke patients after rehabilitation therapy. This enables timely adjustments to the rehabilitation plan (105, 106).

## 3 Discussion

### 3.1 Existing methods

In current Artificial Intelligence-related research, due to clinical practical needs and challenges in data acquisition, researchers primarily utilize CT and MRI imaging modalities to assess ischemic stroke. Among these, MRI is undoubtedly the most widely used imaging modality. Although MRI has certain limitations for patients with metallic implants or claustrophobia, it can detect ischemic lesions earlier and more sensitively through diffusion-weighted imaging. The multiple imaging modalities of MRI also enable researchers to acquire more medical information, constructing more robust models. Another primary imaging modality without a doubt is CT. CT, as a simple, feasible, and cost-effective diagnostic method, is widely favored among clinicians and emergency patients. CT enhancement or perfusion imaging methods performed on the basis of routine CT scans have been proven to have a powerful detection and evaluation effect.

Currently, the number of studies based on machine learning and deep learning is roughly equal. In terms of machine learning, modeling based on image texture features remains popular among researchers. Meanwhile, methods for extracting image texture features are applicable to various MRI sequences and facilitate the integration of information from different sequence images. However, this method requires the annotation of regions of interest (ROIs) to ensure consistency, leading to a significant amount of manual annotation and inspection of images, making it difficult to include a large number of samples in this type of research. Additionally, it is noteworthy that there are very few studies in rehabilitation-related machine learning research that adopt entirely new algorithms. Researchers still use several classical machine learning algorithms for modeling and determine the best model after comparing using a single evaluation parameter (usually AUC). Research related to deep learning has been rapidly growing in recent years. Both methods of data annotation are used by researchers. Firstly, studies based on deep learning can, like those based on texture analysis, use only ROIs for classification after re-annotation. This method constrains the data acquisition range through prior knowledge, which can effectively improve the model’s accuracy under normal circumstances. Another method directly uses 3D brain images. Unlike the ROI annotation method, this method greatly expands the source of information. For strokes, this method incorporates not only information related to the severity of the injury within the ROI (usually the lesion) but also extracts information such as the relative extent and spatial location of the damage. This may predict the patient’s recovery status by evaluating potential compensatory capabilities. More importantly, using 3D images to build models eliminates the need for manual annotation, greatly reducing the difficulty of expanding the sample size and helping to improve the robustness of the model. Although neural network structures are more flexible compared to machine learning, researchers still prefer to use neural networks that have shown good performance on other types of datasets, rarely adjusting the neural network structure based on task characteristics. This means that there is still significant room for improvement in stroke rehabilitation research based on deep learning.

### 3.2 Summary

Ischemic stroke can lead to serious consequences, including permanent brain damage and neurological deficits. Therefore, reducing and preventing neural damage caused by strokes has always been a focal point of research. Assessing the long-term and short-term recovery of patients through imaging enables the early identification of those requiring intervention and facilitates adjustments to rehabilitation plans based on imaging evaluation results, thereby enhancing patient quality of life. As one of the non-invasive diagnostic methods capable of acquiring a large amount of medical information, AI-based neuroimaging has demonstrated its ability to assess the long-term and short-term recovery of stroke patients. This suggests that AI-based neuroimaging holds potential for guiding rehabilitation programs. Therefore, conducting neuroimaging follow-up during therapy and using AI methods to clarify the qualitative and quantitative relationships between rehabilitation interventions and neuroimaging is an area worthy of further exploration by researchers.

## Author contributions

ZZ: Writing – original draft. YZ: Writing – original draft. JS: Writing – original draft. LY: Visualization, Writing – original draft. LP: Project administration, Writing – original draft. YG: Visualization, Writing – original draft. HW: Funding acquisition, Supervision, Writing – review & editing.
